# Versatility of Cooperative Transcriptional Activation: A Thermodynamical Modeling Analysis for Greater-Than-Additive and Less-Than-Additive Effects

**DOI:** 10.1371/journal.pone.0034439

**Published:** 2012-04-10

**Authors:** Till D. Frank, Aimée M. Carmody, Boris N. Kholodenko

**Affiliations:** 1 Systems Biology Ireland, University College Dublin, Belfield, Dublin, Ireland; 2 University College Dublin School of Physics, University College Dublin, Belfield, Dublin, Ireland; Center for Genomic Regulation, Spain

## Abstract

We derive a statistical model of transcriptional activation using equilibrium thermodynamics of chemical reactions. We examine to what extent this statistical model predicts synergy effects of cooperative activation of gene expression. We determine parameter domains in which greater-than-additive and less-than-additive effects are predicted for cooperative regulation by two activators. We show that the statistical approach can be used to identify different causes of synergistic greater-than-additive effects: nonlinearities of the thermostatistical transcriptional machinery and three-body interactions between RNA polymerase and two activators. In particular, our model-based analysis suggests that at low transcription factor concentrations cooperative activation cannot yield synergistic greater-than-additive effects, i.e., DNA transcription can only exhibit less-than-additive effects. Accordingly, transcriptional activity turns from synergistic greater-than-additive responses at relatively high transcription factor concentrations into less-than-additive responses at relatively low concentrations. In addition, two types of re-entrant phenomena are predicted. First, our analysis predicts that under particular circumstances transcriptional activity will feature a sequence of less-than-additive, greater-than-additive, and eventually less-than-additive effects when for fixed activator concentrations the regulatory impact of activators on the binding of RNA polymerase to the promoter increases from weak, to moderate, to strong. Second, for appropriate promoter conditions when activator concentrations are increased then the aforementioned re-entrant sequence of less-than-additive, greater-than-additive, and less-than-additive effects is predicted as well. Finally, our model-based analysis suggests that even for weak activators that individually induce only negligible increases in promoter activity, promoter activity can exhibit greater-than-additive responses when transcription factors and RNA polymerase interact by means of three-body interactions. Overall, we show that versatility of transcriptional activation is brought about by nonlinearities of transcriptional response functions and interactions between transcription factors, RNA polymerase and DNA.

## Introduction

Combinatorial regulation of gene expression involves different receptor ligands, signaling pathway crosstalk, and different transcription factors. Such a combinatorial regulation can give rise to both synergistic activation responses [1], [2] and responses similar to Boolean switches such as AND and OR gates [3], [4]. For the special case of multiple transcription factors regulating gene expression the term ‘cooperative transcriptional activation’ has frequently been been used [5]–[8]. This cooperative activation can induce gene expression levels that are significantly higher than the naively expected ‘additive’ gene expression levels obtained by summing up the transcription rates induced by individual transcription factors. This phenomenon is referred to as ‘greater-than-additive effect’.

**Figure 1:**
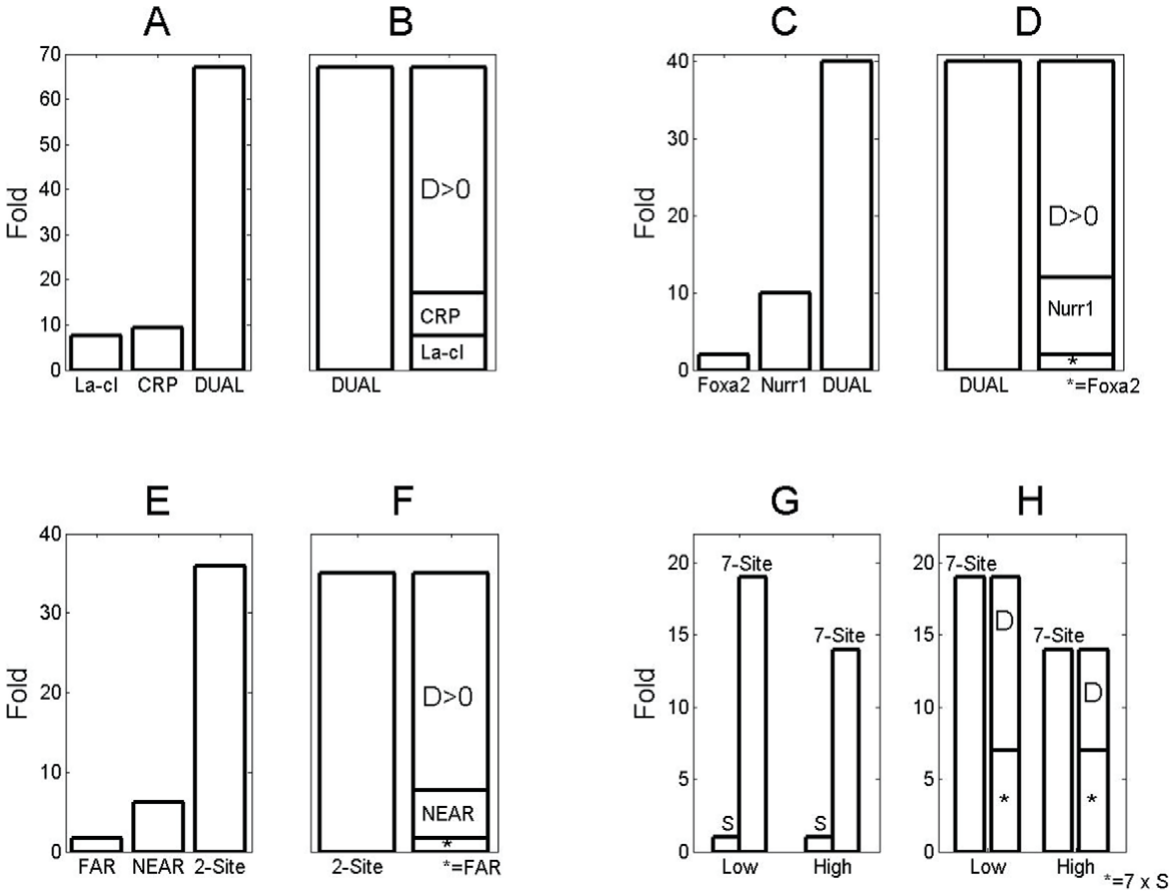
Illustration of transcriptional greater-than-additive responses reported in the literature. Bars labeled ‘D’ represent the magnitude of a reported greater-than-additive effect. Panels A–B, C–D: experiments involving two different transcription factors. Panels E–F, G–H: experiments involving promoters with more than a single binding site for the same transcription factor. A–B: study by Joung et al. (1994) on a promoter stimulated by 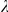
cI and CRP. C–D: From a study Lee et al. (2010) that illustrates the cooperative activation by means of Foxa2 and Nurr1. E–F: In another study by Joung et al. (1993) greater-than-additive responses were observed when comparing a synthetic promoter with two CRP binding sites with a promoter exhibiting only a single CRP site. G–H: Chi and Carey (1996) compared transcriptional activity from promoters with a single ZEBRA binding site and seven ZEBRA sites.

Cooperative activation exhibiting greater-than-additive effects can involve different species of transcription factors or several transcription factor molecules of the same type, as illustrated in Figure 1. For example, Joung et al. studied the synergistic activation of transcription by means of the bacteriophage 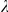
cI protein and the *E. coli* cyclic AMP receptor protein (CRP) [6]. To this end, an artificial promoter was constructed with a binding site for 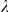
cI activator relatively close to the core promoter (transcription start site) and a binding site for the CRP transcription factor upstream of the transcription start site. Stimulation by means of 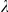
cI and CRP produced a greater transcriptional activity than the sum of the transcriptional activities as induced by individual stimulations via 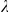
cI and CRP. In Figure 1A the fold changes reported in [6] for the respective stimulations are shown. Here the label ‘DUAL’ refers to stimulation of transcription by means of both 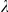
cI and CRP. In Figure 1B the individual responses to 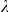
cI, on the one hand, and CRP, on the other, are summed up and the result is compared with the transcriptional response of the dual (combined) stimulation. The discrepancy or difference is illustrated as an additional bar labeled 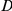. Obviously, 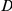 is positive. That is, Joung et al. illustrated that the two transcription factors, 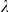
cI and CRP, can produce a greater-than-additive response, at least within the framework of the aforementioned artifical promoter. Similarly, Lee et al. [9] reported cooperative transcriptional activation by the orphan nuclear receptor transcription factor Nurr1, and Foxa2, a transcription factor belonging to the forkhead box family. Lee et al. reported a greater-than-additive effect of combined stimulation involving Nurr1 and Foxa2 on tyrosine hydroxylase (TH) expression levels. Figure 1C depicts the fold changes in gene expression observed in this study, while Figure 1D illustrates the greater-than-additive effect (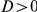). To investigate how Nurr1 and Foxa2 cooperatively regulate TH expression is an important matter because in related studies it has been argued that Nurr1 regulates TH expression [10]–[12] but it is also known that Foxa2 controls the development of TH expressing cells (see e.g. Ref. [13]).

As mentioned above, a greater-than-additive response to cooperative stimulation may also be found when several transcription factor molecules of the same type are bound at different promoter sites. In addition to the aforementioned experiments by Joung et al., in a separate study [5] they constructed promoters with two binding sites for the transcription factor CRP. Similar synthetic promoters with CRP sites were also engineered by Busby et al. [14]. We will refer to the two binding sites addressed in these two studies as ‘near’ and ‘far’ binding sites, where ‘near’ corresponds to the binding site located relatively close to the transcription start site and ‘far’ corresponds to the second binding site located further upstream. In the studies by Joung et al. and Busby et al. it was found that the double binding site promoters induced a transcriptional activity that is larger than the sum of the activities recorded from the respective two types of single binding site promoters (single ‘near’ site or single ‘far’ site). Figure 1E depicts the transcriptional activities from the Joung et al. study as measured in fold changes for the three conditions: only the ‘far’ site is active, only the ‘near’ site is active, and both sites are active. From the construction in Figure 1F it is clear that Joung et al. observed a greater-than-additive effect (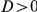). Likewise, Chi and Carey studied the cooperative impact of trans-acting ZEBRA proteins [15]. Chi and Carey recorded transcriptional activity from two different promoters, the first promoter exhibiting only a single ZEBRA binding site, the second featuring seven binding sites. As shown in Figure 1G, transcriptional activity was higher for the promoter with 7 binding sites. However, the observed activity was even higher than the hypothetical value assuming an additive model (i.e., it was higher than 7 times the transcriptional activity of the single-ZEBRA-site promoter), thus exhibiting a greater-than-additive effect (see Figure 1H). Overall, Chi and Carey observed a greater-than-additive effect. Interestingly, the effect was dependent on the concentration of the trans-acting factors. The magnitude of the effect decreased when the magnitude of the stimulation was increased, see Figure 1H again (the 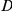 bar in the low-dose condition is larger in magnitude than the 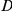 bar in the high-dose condition). These results by Chi and Carey were consistent with results obtained in other studies [16], [17].

Note that a plentitude of experimental studies have been conducted that report cooperative activation in general, and in particular greater-than-additive effects. The aforementioned examples represent only a few such studies. Since greater-than-additive responses have been frequently highlighted in the literature, it is important to consider the mechanisms leading to such responses and to support the plausibility of those mechanisms by means of quantitative models. In fact, it has been argued that greater-than-additive effects are caused by at least two different mechanisms [18]. On the one hand, the nonlinear (sigmoidal) characteristics of the transcriptional machinery may result in greater-than-additive effects. On the other hand, there are instances in which multiple transcription factors can initiate transcription by mechanisms that may not be available to single transcription factors (e.g., looping of DNA or the assembly of activation complexes). As far as the quantitative modeling of cooperative transcriptional activation is concerned, various statistical modeling approaches have been developed [7], [8], [19]–[28]. Such statistical modeling efforts in general involve two steps. Firstly, a set of mutually exclusive DNA states (or DNA configurations) is identified for the problem under consideration. Secondly, the probability of observing a particular state when randomly selecting a cell out of a cell population is determined. To this end, thermostatistical arguments [7], [8], [19]–[22], [24], [26], [29], [30] have been used, in particular in combination with reaction kinetics approaches [22], [31], [32].

Of particular interest are DNA states where RNA polymerase is bound at the promoter, thereby initiating transcription. The cumulative probability obtained from all these states provides a general measure for gene expression and in particular for transcription initiation [7], [8], [19]–[22].

Statistical approaches have the benefit of allowing us to derive mathematical expressions for transcription rates without introducing levels of complexity that are not well understood and go beyond the identification of transcription factors and transcription factor binding sites. In particular, analytical expressions for transcription rates can be obtained with predictive power and in doing so can guide the design of experimental studies. However, there is still a demand for the characterization of the key features of thermostatistical models of cooperative transcriptional activation. The reason for this is that by definition the models are defined on multi-dimensional state spaces, which is a key challenge to a rigorous and systematic analysis (and implies considerable computational efforts for parameter estimation) [24], [32]–[34].

In previous studies, focus has primarily been on the binding probabilities of transcription factors, while a statistical treatment of the binding of the RNA polymerase has been neglected [32], [34], [35]. In contrast, our approach will address the binding probability of RNA polymerase explicitly and in doing so our modeling approach will admit for a discussion of basal transcription rates. Likewise, some previous studies have primarily focused on multiple transcription factors acting individually on RNA polymerase [3], [29], [30], [36]. Since such interactions of individual transcription factors and RNA polymerase include only a particular transcription factor and the RNA polymerase molecule, they will be referred to as two-body interactions. Our thermostatistical modeling approach will generalize the two-body interaction case to interactions of higher order. Such higher-order interactions have previously been studied by means of model-based approaches for promoters featuring several binding sites for the same transcription factor [22]. As opposed to these previous efforts, we are interested in studying interactions between RNA polymerase and two transcription factors (three-body interactions) that are not necessarily identical to each other. In this context, an issue is to distinguish between the effects of two-body and three-body interactions.

We will present a general statistical model for cooperative activation by means of an arbitrary number of transcription factors below (Section Methods). The derivation can be found in Text S1. Subsequently, we will illustrate this model for the important special case of transcriptional regulation by means of two activators. The Results section is dedicated to synergistic effects and less-than-additive effects. The latter are the negation of greater-than-additive effects. In the subsection ‘Greater-than-additive and less-than-additive effects’ conditions will be derived under which these effects can be observed. In the subsection ‘Cross-over behavior induced by the dose increase of transcription factors’ we determine cross-over points at which less-than-additive responses to transcriptional activation patterns turn into greater-than-additive responses. Both dose-induced transitions from less-than-additive to greater-than-additive responses and greater-than-additive to less-than-additive responses will be addressed. The latter involve a decrease of the magnitude of the greater-than-additive response as observed by Chi and Carey (see Figure 1H) and occur in the context of so-called re-entrant transitions.

Such re-entrant phenomena, in turn, are well known in physics [37]–[41] (see also Sec. 7.3 in Ref. [42]). Roughly speaking, a system parameter is scaled up gradually while passing two critical threshold values. At the first threshold the system's state, behavior, or response pattern changes qualitatively from state A to B. At the second threshold, the system switches back from B to A. Re-entrant phenomena are crucially important for our understanding of complex systems, in general, and biological systems, in particular, because they indicate that the system under consideration must be fine-tuned [43] in order to be able to exhibit the behavior B rather than the alternative behavior A.

## Methods

### Presentation of the general thermostatistical model: multiple transcription factors

Let us consider 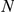 transcription factors 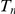, 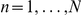 that regulate the transcription of a particular gene by binding to specific sites in the regulatory region of the DNA. Consequently, each transcription factor binding site can be observed in two conditions: occupied or not. Likewise, RNA polymerase (RNAP) is described by a binary variable since RNAP can be bound to the promoter or not. In the former case the promoter is activated and transcription is initiated. In general, the transcriptional machinery exhibits different configurations or states. We assume that there are 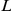 states of interest. By convention, the state 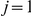 corresponds to DNA with a regulatory region free of RNA polymerase and transcription factors (i.e., neither RNA polymerase nor transcription factors are bound). The state 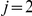 corresponds to DNA with RNA polymerase bound to the promoter without any transcription factor involved. In general, each state 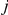 is described by a chemical reaction. For example, for the aforementioned DNA state 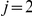 the chemical reaction reads
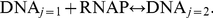
(1)Consequently, DNA states 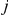 are described by reaction equations of the form
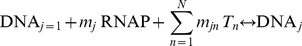
(2)for 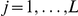, where 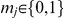 is a vector, 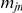 is a matrix of stoichiometric coefficients, and 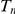 are the aforementioned transcription factors 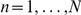. Here 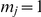 (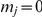) indicates that in the state 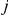 RNA polymerase is (is not) bound to the promoter.

Our objective is to determine the probability 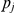 when selecting randomly a cell out of a population of cells to find the DNA of that cell in the state 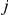. Let 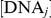 denote the concentration of cells in DNA state 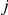. Then, the probability 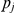 of observing a randomly selected cell in a DNA state 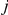 is defined by [31]

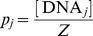
(3)with the partition function 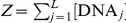. These probabilities depend in general on the concentrations 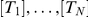 of the transcription factors 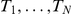 and on the concentration of RNA polymerase 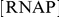. Moreover, the binding probabilities depend on various parameters describing the regulatory impacts of the transcription factors and the interactions between transcription factors and RNA polymerase. As shown in Text S1, the probabilities 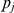 are explicitly given by
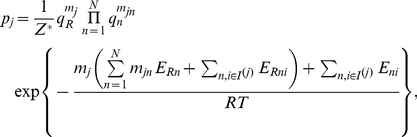
(4)where 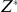 is related to 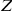 (see Text S1) and has to be chosen such that the probabilities 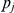 are normalized. In Eq. (4) we have introduced the dimensionless, relative concentrations
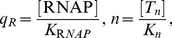
(5)where 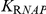 and 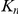 denote the respective dissociation constants (see Text S1 for precise definitions). The parameters 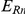, 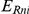, and 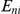 describe shifts of the free energy due to various impacts of transcription factors. Activators lower the binding energy of RNAP by a certain amount. Such energy shifts will be denoted by 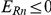. Two transcription factors may affect the RNAP binding energy by mechanisms that cannot be induced by single transcription factors alone. Energy shifts induced by such mechanisms will be denoted by 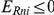. In addition, interactions between transcription factors that do not involve RNA polymerase may affect the free energy. We account for such interactions by introducing energy shift terms denoted by 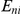. The index-sets 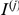 occurring in Eq. (4) describe all transcription factors that are involved in the state 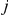 (see also Text S1 for a rigorous definition). Finally, in the exponential function of Eq. (4) the variable 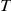 is temperature and 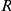 is the Boltzmann gas constant.

Eq. (4) is nonlinear with respect to the energy shifts 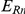. Due to this nonlinearity, several transcription factors can induce a synergistic greater-than-additive effect even if each transcription factor acts only individually on RNA polymerase [18],

A more concise description of the DNA state probabilities 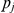 can be obtained by means of the variable transformation
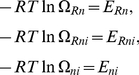
(6)that relates the energy variables 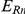, 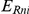, and 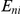 to a set of 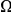-parameters with 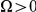 for all 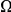-parameters. The parameters 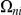 and 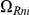 are referred to as cooperativity factors because they describe the interaction between two transcription factors (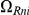) or two transcription factors and RNA polymerase (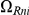). By virtue of Eq. (6) the thermostatistical model (4) can be cast into the form
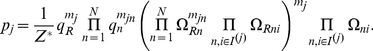
(7)


The probability 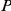 that RNAP is bound at the promoter is given by
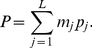
(8)This is the probability to find cells with an activated promoter. For our purposes, it is useful to express this probability in an alternative way, by introducing the total relative concentrations of ‘on’ and ‘off’ states:
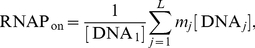
(9)

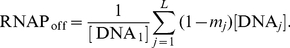
(10)

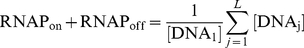
(11)Note that by convention we have normalized these concentrations to the concentration 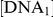 of cells with DNA that exhibits neither bound transcription factors nor bound RNAP. By definition, we have 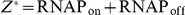 and [3], [26]

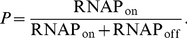
(12)


### Transcriptional regulation by two activators

Our next objective is to study gene expression regulated by two transcription factors using the statistical approach outlined above. We refer to the two transcription factors as 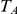 and 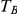 rather than 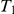 and 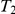. For two transcription factors that can be bound or not bound to the DNA, the transcriptional machinery exhibits 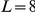 possible states. These states are listed in Table 1. In Table 1 we also list the free energy shifts 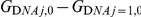 for one standard unit that are related to the energy shifts in Eq. (4) and are defined by Eq. (7) of Text S1. Moreover, Table 1 lists the relative equilibrium concentrations 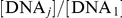, which are proportional to the binding probabilities 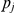 and are defined explicitly by Eq. (13) in Text S1.

**Table 1:** Characteristic features of a statistical transcriptional activation model with two transcription factors.

State 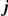	RNAP	TFA	TFB	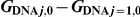	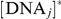
1	-	-	-	0	1
2	x	-	-	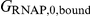	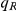
3	-	x	-	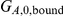	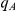
4	-	-	x	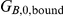	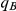
5	x	x	-	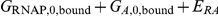	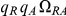
6	x	-	x	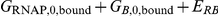	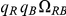
7	-	x	x	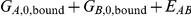	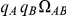
8	x	x	x	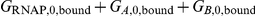	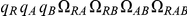
				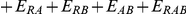	

Characteristic features of the statistical model for transcription initiation in the case of two transcription factors: states, changes in DNA standard free energies, and state-specific DNA fractions. Here x and - denote bound and unbound forms.

The probability 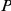 that RNAP occupies the promoter is given by 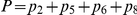. In particular, 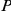 can be computed from Eq. (12) with
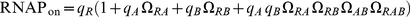
(13)describing events in which RNA polymerase occupies the promoter and
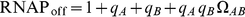
(14)describing events in which RNAP is not bound to the promoter. Explicitly, we obtain
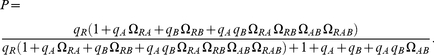
(15)Note that Eq. (12) can alternatively be expressed by means of the regulatory function as suggested by Bintu et al. [7], [8] (see Text S1). It has frequently been assumed that the transcription rate 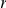 of a protein is proportional to the binding probability 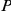 [7], [8], [20]–[22], [24], [44]. Accordingly, we put
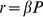
(16)with 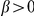. It can be shown (see Text S1) that the probability 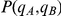 and consequently the transcription rate 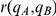 increases monotonically in both directions 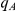 and 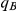, i.e., we have
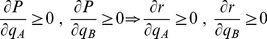
(17)for 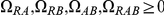. This implies that the mathematical expressions (15) for the RNAP binding probability and (16) for the transcription rate are consistent with the fundamental notion of activators in the sense that when activator concentrations are scaled up then binding of RNAP is supported and transcriptional activity increases.

## Results

### Greater-than-additive and less-than-additive effects

We define the difference
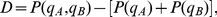
(18)which is a function of the relative activator concentrations 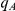 and 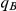 but also depends on the quantities 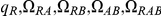. If 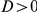 (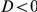) we have a greater-than-additive (less-than-additive) effect. In applications to biological data we may distinguish between two situations

- Transcription factor concentrations are varying. In this case 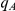 and 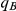 are considered as variables and 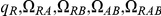 are parameters.
- We compare different (mutant) promoters under the same type of stimulation (e.g., saturation). In this case, 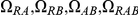 may be considered as variables and 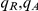, and 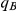 may referred to as parameters.

In general, the difference measure 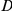 is defined on the vector space spanned by the seven dimensional vector
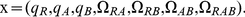
(19)By means of a detailed, mathematically analysis (see Text S1) domains in this vector space can be identified, where greater-than-additive and less-than-additive effects are predicted by the statistical model (15). The results are summarized in Table 2.

**Table 2:** Greater-than-additive and less-than-additive effects, their domains, and causes.

Cases	Key feature	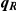	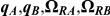	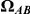	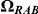	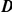
	(Causes)					
1	Low TF concentrations	—	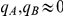	—	—	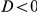
	e.g., weak stimulations					
2	Weak activators	—	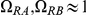	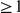	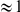	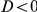
3	High RNAP	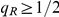	—	1	1	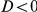
	concentrations					
4	Weak activations	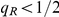	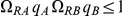	1	1	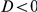
5a	Nonlinearities &	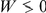	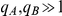	1	1	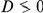
	high TF concentrations		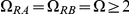			
	e.g., strong stimulations					
5b	Nonlinearities &	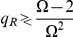	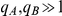	1	1	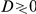
	high TF concentrations		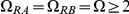			
	e.g., strong stimulations					
6	Nonlinearities &	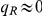	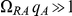	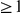	1	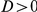
	strong activations		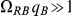			
			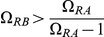			
7a	Three-body	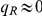	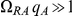	1	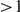	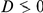
	interactions		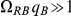			
			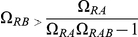			
7b	Strong	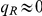	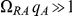	1	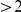	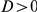
	three-body		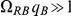			
	interactions					

Summary of cases in which the thermostatistical model predicts greater-than-additive and less-than-additive effects involving particular key features. These features may be regarded as causes of the associated greater-than-additive or less-than-additive effects. ‘TF’ stands for ‘Transcription factor’. The function 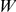 is defined by Eq. (20).

#### Low transcription factor concentrations and weak activators

At low concentrations of transcription factors, i.e., for 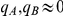 cooperative activation by means of two transcription factors can only produce less-than-additive responses (case 1 in Table 2). Such low transcription factor concentrations may be due to weak receptor signals (weak stimulation). Likewise, when activators only induce relatively small energy shifts 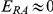, 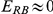, 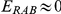 (weak activators), then the transcriptional machinery exhibits only less-than-additive responses even if the two activators can lower their binding energy due to are relative high interaction energy 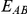 (case 2). These two cases imply that synergistic greater-than-additive effects must emerge from less-than-additive effects when scaling up transcription factor concentrations or when replacing weak activators by stronger ones.

#### Nonlinearities of the thermostatistical transcriptional machinery

As mentioned above, Eq. (4) is nonlinear with respect to the energy shifts 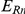. Consequently, two transcription factors may induce greater-than-additive effects even if each transcription factor acts only individually on RNA polymerase, i.e., even if 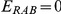. We examined this case in more detail for 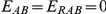 (i.e., 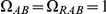). As illustrated in Table 2, the thermostatistical model predicts that at relative high RNA polymerase concentrations nonlinearities cannot contribute to synergistic greater-than-additive effects. More precisely, when the RNAP concentration is greater than half of its dissociation constant (i.e., 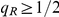) and 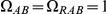 holds, then cooperative stimulation by means of two activators yields only less-than-additive effects.

**Figure 2:**
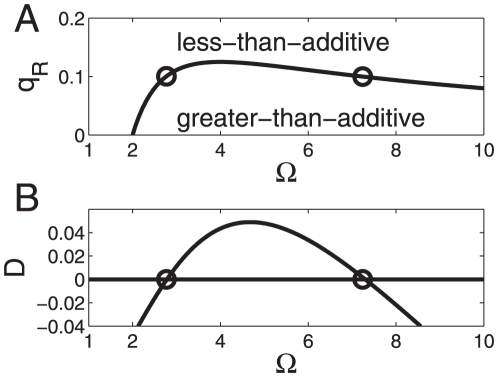
Parameter domains for greater-than-additive and less-than-additive responses to saturated stimuli. Parameter space 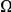-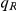 is considered here. Cooperative effects are caused by the nonlinearity of the RNA polymerase binding probability function (15). (A) The function 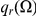 was computed from Eq. (21). For all parameter values 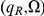 on that function the transcriptional machinery exhibits additive responses (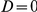). Critical values of 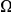 for 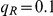 are indicated by circles and correspond to the circles shown in panel B. (B) The function 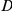 as computed from Eqs. (15,18) as a function of the strength of the transcription factor impact 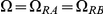. When 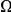 is increased a re-entrant transition can be observed. Parameters for panel B: 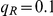, 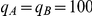, 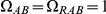.

**Figure 3:**
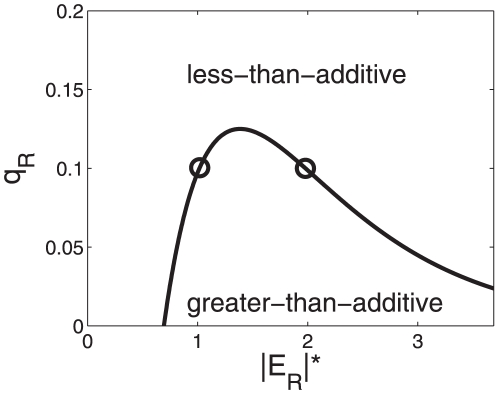
As in **Figure 2A** but in the space space 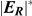
 **-** 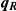 **rather than** 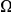 **-** 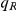 **.** The function 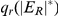 was calculated from Eq. (22).

The situation changes when 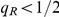. Let us refer to the product 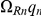 of the energy shifts 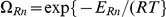 and the relative concentrations 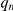 with 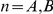 as transcriptional activation due to the transcription factor 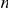. The activation may be low 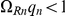 because the activator is weak (i.e., 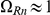) and/or the transcription factor concentration is low (e.g., 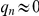). For combined low activation, i.e., for 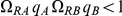, it can be shown that stimulation by means of two transcription factors yields less-than-additive responses (see case 4, Table 2). In contrast, at relatively high transcription factor concentrations the response can be less-than-additive as well as greater-than-additive (cases 5a and 5b). In this context, the sign of the function
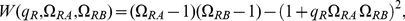
(20)which is quadratic in 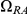 and 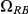, is of crucial importance because 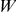 has the same sign as the difference measure 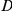 (see Text S1). First note that the two terms 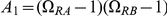 and 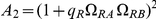 are positive. Second, note that if 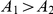 we have 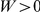 and 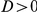. In this case, the combined stimulation with two transcription factors results in a greater-than-additive response. In contrast, for 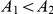 we have 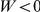 and 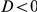 and the thermostatistical model predicts a less-than-additive effect. If the energy shifts 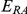 and 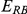 of the two transcription factors are comparable such that 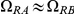 then the domains for less-than-additive and greater-than-additive effects can be determined more precisely (see case 5b in Table 2 and the Text S1). Let us put 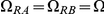. In this case, the seven dimensional space spanned by the vector
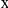
reduces to the two dimensional plane spanned by 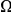 and 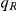 (see Text S1). The critical boundary line 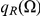 in this space is defined by
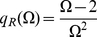
(21)for 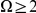. The function is shown in Figure 2A. The function 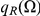 increases monotonically from 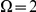 to 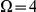 and then decays monotonically. The peak at 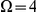 is 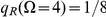 (and is smaller than 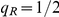 as expected from case 3 of Table 2; we will return to the 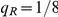 threshold below in the section on cross-overs behavior). In the subspace between the graph 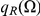 and the 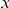-axis (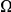-axis) the transcriptional machinery exhibits synergistic nonlinearity-induced greater-than-additive effects when stimulated cooperatively by two transcription factors. In the 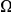-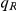 subspace above the graph 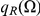 only less-than-additive effects can be observed. When we scale up the parameter 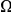 then a re-entrant transition from a less-than-additive response to a greater-than-additive response and back again to a less-than-additive response is found, see Figure 2B. That is, for small energy shifts 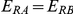 less-than-additive effects are predicted, for medium shifts greater-than additive effects are predicted, whereas for large shifts again less-than-additive effects are predicted. In particular, using 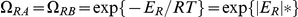 with 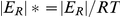, Eq. (21) becomes
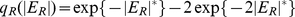
(22)for 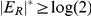, see Figure 3. Figures 2 and 3 illustrate that for a given relative RNAP concentration 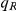 in order to produce a greater-than-additive response the system must be fine-tuned [43] (at least to some degree) with respect to the system parameter 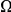 and/or energy shift 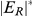.

**Figure 4:**
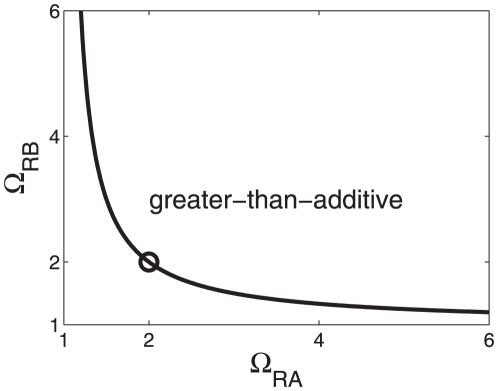
Parameter domain in the space 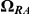-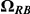 for greater-than-additive responses to strong activations. Cooperative effects are caused by the nonlinearity of the RNA polymerase binding probability function (15) and may (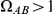) or may not (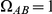) be affected by two-body interactions between transcription factors. The hyperbola 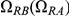 was computed from Eq. (23). In general, the hyperbola defines a critical line such that greater-than-additive effects are predicted for parameters values 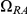, 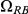 ‘above’ that line. In particular, the parameter conditions 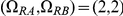 described by the circle were analyzed in Figure 5 in more detail assuming that 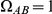 holds. Strong activation: 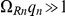, 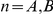. Depending on the values of other transcription-relevant parameters, the transcriptional machinery may or may not exhibit additive responses for parameters 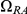, 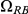 on that line. For example, for 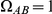 it follows (by comparison with the construction in Figure 6) that 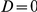 holds on the hyperbola and 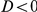 holds in the area ‘below’ the hyperbola.

**Figure 5:**
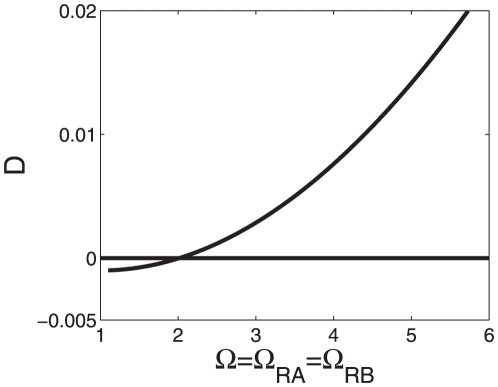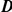 as a function of 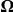 for strong activations. The function 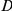 was computed from Eqs. (15,18) for the transcription factor impact 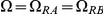. The transcriptional machinery exhibits greater-than-additive effects for 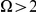 as predicted from the hyperbola shown in Figure 4. In fact, the function 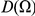 was computed for 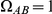, which implies that the model predicts for 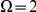 an additive response and for 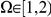 a less-than-additive response. Parameters: 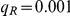, , 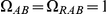.

Let us close our considerations on the impacts of nonlinearities of the thermostatistical transcriptional machinery. To this end, we examine gene expression involving relatively small transcription rates. In Ref. [7], [8] this case has been used to test whether or not transcription factors act independently on the promoter. We assume that 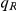 is small which implies that the RNAP binding probability 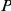 and consequently the transcription rate 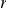 are small quantities as well (see Eqs. (15) and (16)). Moreover, we focus on relatively strong activations, i.e., we assume that the products 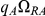 and 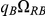 satisfy 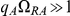 and 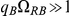. Note that the products can be large because the transcription is subjected to high relative transcription factor concentrations 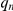 and/or transcription involves strong activators with 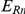 large. For 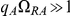 and 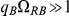 it can be shown that only the projection of the seven dimensional space 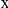 to the subspace given by 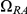 and 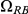 is relevant in order to identify conditions for a synergistic greater-than-additive response (see Text S1). In particular, in the 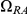-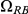 space the hyperbola function
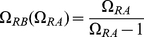
(23)shown in Figure 4 defines a critical line. For two activators 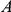 and 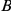 that induce sufficiently large energy shifts 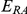 and 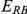, respectively, i.e., exhibit parameters 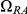 and 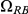 that correspond to points 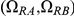 located ‘above’ the hyperbola (23), we conclude that the combined stimulation by means of 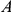 and 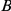 results in a greater-than-additive effect (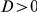), see also Table 2 (case 6). Figure 5 shows how the difference 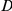 becomes positive when we increase 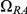 and 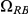 along the diagonal, i.e., for 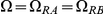. As predicted by the hyperbola shown in Figure 4, we see in Figure 5 that for 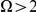 the difference 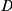 becomes positive.

#### Three-body interactions

**Figure 6:**
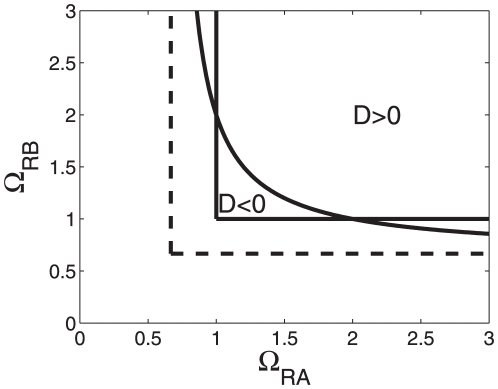
Parameter domains in the space 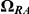
 **-** 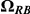 **for** 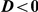 **and** 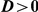 **responses to strong activations.** Cooperative effects are caused by three-body-interactions between RNA polymerase and the transcription factors 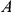 and 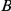 (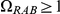) that modify the nonlinear characteristics of the RNA polymerase binding probability function (15). Two-body-interactions between the two transcription factors are assumed to be negligible (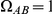). The function 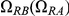 was computed from Eq. (24). 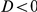 indicates less-than-additive responses. 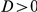 indicates greater-than-additive responses. Solid lines indicate the parameter domain relevant for activators. Dashed lines indicate locations of asymptotes. Parameter: 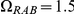. Strong activation: 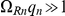, 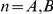.

The energy shifts 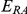 and 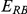 describe how the transcription factors 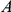 and 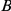 lower independently from each other the RNA polymerase binding energy and in doing so increase the rate of transcription initiation and eventually increase the rate of protein transcription. In contrast, the energy shift 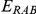 describes that the transcription factors 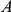 and 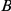 act together (e.g., via looping [7], cooperative binding [2], [22], cooperative attraction of adapter factors [33], etc) such that the binding probability of RNA polymerase increases. A detailed analysis of the thermostatistical model for transcription initiation reveals that under certain circumstances this type of three-body interaction yields a greater-than-additive effect (see Table 2, cases 7a and 7b; see also Text S1). More precisely, we consider strong activations 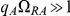, 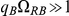 given low RNAP concentrations (i.e., 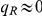). The latter implies that the following discussion applies to gene expression at relatively low transcription rates 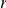. In this case, we can distinguish between promoters exhibiting less-than-additive responses and promoters exhibiting greater-than-additive responses by defining the hyperbola
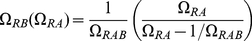
(24)on the two-dimensional plane spanned by 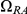 and 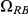 (when 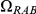 is considered a parameter). The hyperbola is shown in Figure 6 with the asymptotes at 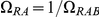 and 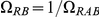. Promoters with activators described by the parameters 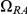 and 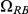 that correspond to a location ‘above’ (‘below’) the hyperbola exhibit greater-than-additive responses (less-than-additive responses) when stimulated by both transcription factors. The domain related to greater-than-additive effects increases when 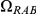 is increased. In particular, the asymptotes 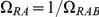 and 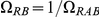 approach the vertical and horizontal axes for 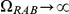.

**Figure 7:**
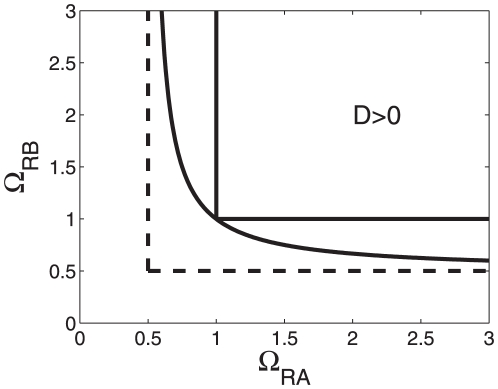
As in **Figure 6** but for 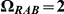 rather than 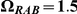. The figure demonstrates that the parameter domain relevant for activators to induce less-than-additive responses disappears for strong three-body interactions 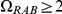.

Note that for 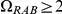 the transcriptional machinery exhibits only greater-than-additive responses to combined stimulation by both transcription factors (see also Table 2, case 7b). The reason for this is that for 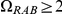 the parameter domain 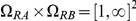 relevant for activators is entirely contained in the area ‘above’ the critical hyperbola 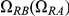 defined by Eq. (24), see Figure 7. We may refer to transcription factors acting on promoters with  as promoters exhibiting ‘strong three-body interactions’.

#### Experimental case studies: illustrations for synergistic activation by means of two different activators

**Figure 8:**
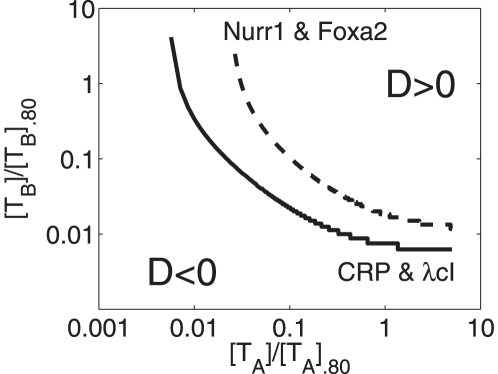
Predicted domains of greater-than-additive and less-than-additive responses for two promoters studied by Joung et al. (1994) and Lee et al. (2010). For rescaled activator concentrations 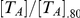 and 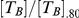 corresponding to a point ‘above’ (‘below’) the hyperbolic lines the thermostatistical model predicts a greater-than-additive (less-than-additive) transcriptional response. Solid line: re-analysis of the study by Joung et al. [6] of a engineered promoter regulated by the transcription factors 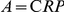 and 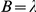
cI. Dashed line: re-analysis of the study by Lee et al. [9] involving a promoter regulated by the transcription factors 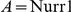 and 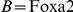.

As mentioned in the introduction and illustrated in Figure 1A–B, Joung et al. studied the cooperative transcriptional activation by means of the bacteriophage 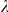
cI protein and the *E. coli* cyclic AMP receptor protein (CRP) [6]. A synthetic promoter was designed with a binding site for the 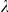
cI activator and another binding site for the CRP transcription factor. The transcriptional response to the stimulation by means of 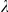
cI and CRP was greater than the sum of the transcriptional responses induced by individual stimulations via 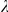
cI and CRP, see Figure 1A–B. Following the suggestion by Bintu et al. [8] from the data of Joung et al. [6] estimates for 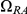, 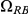, and 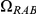 can be obtained, where transcription factors 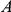 and 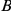 refer to 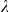
cI and CRP, respectively. We obtained: 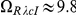, 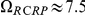, 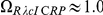. Using Eq. (15) for 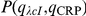 and the corresponding equations for 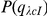 and 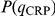, we computed the domains in which 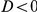 and 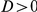 holds and in particular calculated the critical boundary 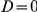 to identify the conditions under which less-than-additive and greater-than-additive effects are predicted by the thermostatistical model. The critical line is shown in Figure 8 (solid line). The transcriptional activities reported by Joung et al. are assumed to reflect saturation values [8] (i.e., we have ). Accordingly, the horizontal and vertical axes shown in Figure 8 reflect transcription factor concentrations 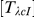 and 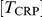 relative to those concentrations that would induce 80 percent of the transcriptional saturation activities found for individual stimulations via 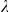
cI and CRP. The artifical promoter used by Joung et al. is then characterized by points located in the top right corner of the two-dimensional plane shown in Figure 8. As mentioned above, greater-than-additive effects must emerge from less-than-additive effects at low transcription factor concentrations. Consequently, our analysis predicts that decreasing the transcription factor concentrations of 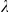
cI and CRP would result in a cross-over from the greater-than-additive effect observed by Joung et al. to a less-than-additive response. Moreover, our model-based analysis provides rough estimates for the critical transcription factor doses of 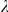
cI and CRP at which the greater-than-additive response would turn into a less-than-additive response (see Figure 8 again). Note that the critical line in Figure 8 looks similar to the hyperbolic lines shown in Figures 6 and 7. However, Figure 8 shows domains of less-than-additive and greater-than-additive effects in a subspace of the seven dimensional vector space (19) spanned by transcription factor concentrations, whereas Figures 6 and 7 shown such domains in a subspace spanned by two 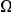 parameters reflecting shifts of the RNA polymerase binding energy.

As summarized in Figure 1C, Lee et al. [9] reported from the cooperative activation of tyrosine hydroxylase by means of the transcription factors Nurr1 and Foxa2. A greater-than-additive response was observed, see Figure 1D. Following the aforementioned methodology of Bintu et al. [8], from the data of the study by Lee et al. we estimated the model parameters 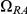, 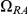, 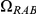, where Nurr1 and Foxa2 represent transcription factors 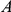 and 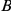, respectively. We obtained: 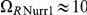, 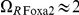, and 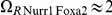. We plotted the boundary line 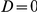 of additive responses in Figure 8 (dashed line). Comparing the promoter studied by Lee et al. with the artifical promoter constructed by Joung et al. we may conclude that the artifical promoter involving the transcription factors 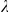
cI and CRP exhibits a larger domain of synergistic activity than the tyrosine hydroxylase promoter activated by Nurr1 and Foxa2.

Having illustrated the applicability of the thermostatistical approach to experimental data, we would like to point out that the aforementioned model-based interpretations are of speculative nature. First, the experiments by Joung et al. and Lee et al. have not been designed to test the thermostatistical model discussed here. Second, as mentioned in the introduction, it is challenge to estimate parameters of thermostatistical models of transcription initiation. The data available in the studies by Joung et al. and Lee et al. do not allow us to determine parameter estimation errors or to conduct model validation methods.

### Cross-over behavior induced by the dose increase of transcription factors

We showed that for low concentrations of transcription factors the RNAP binding probability induced by combined stimulation with both factors is *less than* the sum of the binding probability induced by single activation (less-than-additive effect), see Section ‘Low transcription factor concentrations and weak activators’. We also discussed several scenarios in which the binding probability under combined activation is *larger than* the sum of the binding probabilities induces by individual activations (greater-than-additive effect). These scenarios typically involve large doses of transcription factors (see the Sections ‘Nonlinearities of the thermostatistical transcriptional machinery’ and ‘Three-body interactions’). In order to illustrate the cross-over from less-than-additive to greater-than-additive responses when activator concentrations 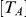 and 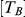 are scaled up, we consider the special case 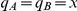 and 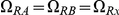. We may consider this simplification just as a mathematical means to allow us to proceed with an analytical rather than a numerical approach. Alternatively, we may consider a promoter with two binding sites 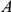 and 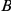 (with identical properties, i.e., binding energies) for a single activator 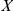 which implies again 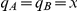 and 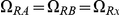.

In short, we put 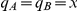 and 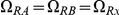 such that the probability (15) becomes
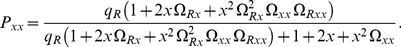
(25)We compare this binding probability with the binding probability of RNAP at a promoter that exhibits only a single binding site for the transcription factor 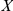. The latter binding probability will be denoted by 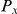. Accordingly, our objective is to demonstrate explicitly that there are critical concentration values 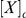 such that for smaller doses 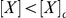 we have 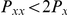, whereas for larger doses 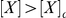 we have 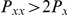.

**Figure 9:**
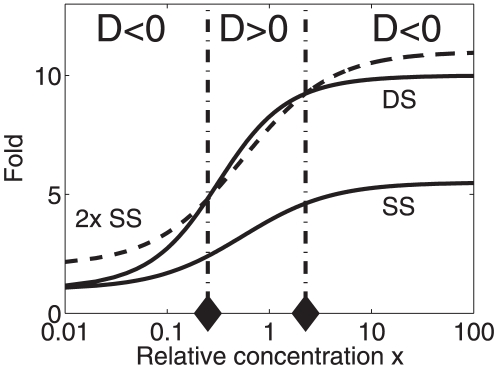
Illustration of a re-entrant dose response predicted by the thermostatistical model for a double-binding-site promoter. The solid line ‘DS’ was computed from Eq. (25) and represents the fold change of the transcriptional activity of a double binding site promoter for a given relative transcription factor concentrations 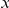. The solid line labeled ‘SS’ represents the transcriptional activity of a corresponding single binding site promoter (as computed from Eq. (82) given in Text S1). The dashed line represents two times the the activity calculated for the single binding site promoter. Diamonds indicate the critical transcription factor concentrations 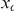 calculated from Eq. (91) in Text S1. As expected, these critical value correspond to the critical values that can be obtained directly from the intersections of the ‘2× SS’ and ‘DS’ graphs. Parameters: 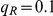, 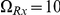. 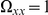, and .

First, we focus on the role of energy shifts 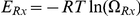, see Eq. (6), and assume that both copies of the transcription factor 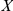 act independently from each other. Accordingly, we study the impact of the nonlinearities of the thermostatistical transcriptional machinery and neglect interactions between transcription factors (i.e., we put 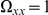) and three-body interactions (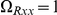). The energy shifts 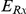 determine the type of transcriptional activity response to a gradually increasing transcription factor dose 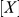 (see Text S1). There are three dose-response patterns: (i) less-than-additive, (ii) single cross-over from less-than-additive to greater-than-additive, and (iii) re-entrant behavior involving a switch from the less-than-additive response to the greater-than-additive response and back to the less-than-additive response. Table 3 summarizes the conditions under which the response patterns can be observed. If the relative RNAP concentration exceeds a threshold concentrations of 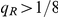, only less-than-additivity is possible for any parameter values 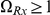 and relative transcription factor concentrations 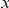. This is consistent with the 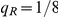 threshold reported above in the section ‘Nonlinearity of the thermostatistical transcriptional machinery’. For 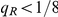 gene expression exhibits the aforementioned patterns (i),(ii), (iii) of dose responses under particular conditions specified in Table 3. The re-entrant behavior (case iii) is exemplified in Figure 9. There are two critical concentrations 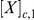 and 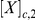 with 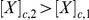. At low transcription factor concentrations (i.e., 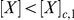), there is a less-than-additive response: 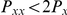 (i.e., 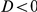). At intermediate concentration levels, 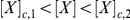, there is a greater-than-additive response: 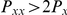 (i.e., 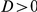). However, at high transcription factor concentrations (i.e., 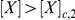) gene expression induced by the double-binding-site promoter exhibits again a less-than-additive characteristics relative to the single-binding-site promoter: 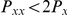 (i.e., 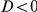). We will return to the re-entrant case in the section ‘Discussion’ in the context of experiments conducted by Chi and Carey [15].

**Table 3:** Three types of dose-response-patterns caused by nonlinearities of the thermostatistical transcriptional machinery for promoters with two identical binding sites for one transcription factor 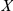.

Type	Response pattern	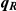	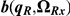	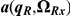
(i)	Less-than-additive	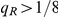	—	—
(i)	Less-than-additive	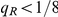	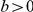	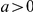
(ii)	Single cross-over	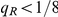	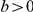	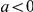
(ii)	Single cross-over	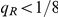	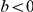	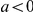
(iii)	Re-entrant	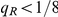	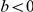	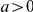

The thermostatistical model of transcription initiation predicts different dose response patterns of gene expression that are caused merely by difference in the nonlinearity parameter 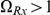 of the thermostatistical transcriptional machinery. The patterns can be observed under conditions that can conveniently be expressed by means of the effective parameters 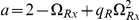 and 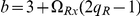. ‘Single cross-over’ means a cross-over from a less-than-additive response to a greater-than-additive response when transcription factor concentrations are scaled up. ‘Re-entrant’ stands for a sequence of less-than-additive, greater-than-additive, and less-than-additive responses. Further parameters: 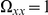 and 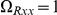.

**Figure 10:**
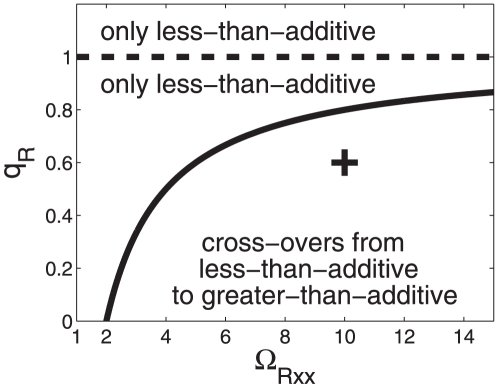
Parameter conditions for observing transitions between less-than-additive and greater-than-additive responses. Less-than-additive and greater-than-additive effects are caused by three-body-interactions between RNA polymerase and two transcription factor molecules of the same type 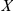 bound at two different promoter sites. Two-body-interactions between the two transcription factor molecules are assumed to be negligible. The function 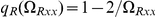 is shown (solid line). The dashed line indicates the asymptote of the solid line.

In the aforementioned discussion we focused on the role of the nonlinearities of the thermostatistical transcriptional machinery. Next, we shift our focus to the three-body interaction between RNA polymerase and the transcription factors bound at the two promoter binding sites. Accordingly, we put 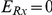 and 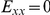 (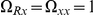) and examine the impact of the energy shift 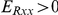 (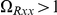). A detailed calculation (see Text S1) shows that transcription exhibits a cross-over from a less-than-additive to greater-than-additive response at a critical dose 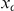 defined by
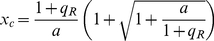
(26)with
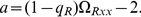
(27)The critical value (25) depends on 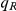 and 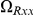 and only exists for parameters 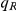 and 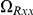 such that 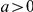. That is, for 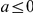 the transcriptional activation exhibits a less-than-additive effect for any relative activator dose 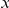. In contrast, if 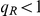 and 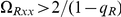 then we have 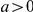 and for small doses 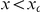 transcription of a double-binding-site promoter shows a less-than-additive effect, whereas for 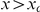 the double-binding-site promoter exhibits a greater-than-additive transcriptional activity relative to the single-binding-site promoter. Figure 10 illustrates the subspace in which the inequalities 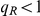 and 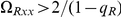 hold. For the sake of consistency with Figure 2, we put 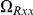 and the horizontal axis and 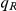 on the vertical axis. That is, in Figure 10 we plotted the critical line 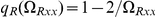 rather than 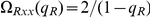.

**Figure 11:**
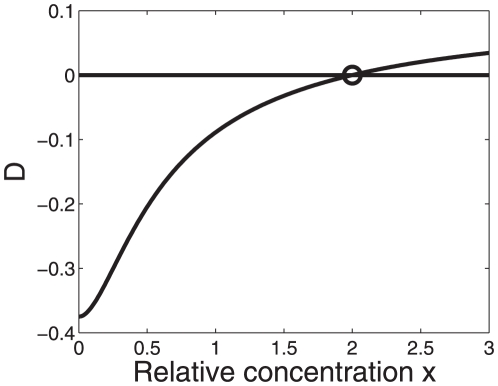
A dose response of a promoter exhibiting 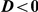
 **and** 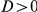 **domains due to three-body interactions.** The function 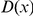 was computed from Eqs. (15,18) for 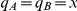, 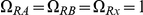, 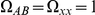, and 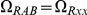. Parameters: 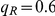 and 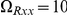. In fact, we also calculated the critical value of 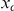 from Eq. (26). The critical value of 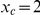 (indicated by the circle) was consistent with the intersection point of 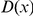.

Figure 11 illustrates the cross-over behavior for an example. We calculated 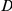 as a function of 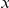 using Eqs. (15,18) with 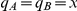, 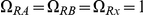, 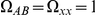, and 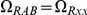. We used the parameters 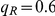 and 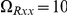 (corresponding to the location indicated by the ‘+’ sign in Figure 10). We found that the function 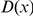 intersects the horizontal axis at 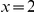. That is, in this example, the transcriptional machinery exhibits less-than-additive responses to transcription factor stimuli with relative doses 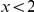 and shows greater-than-additive effects to stimuli with relative doses 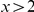. In fact, we also calculated the critical value of 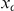 from Eq. (26). We obtain a critical value of 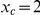 (indicated by the circle in Figure 11) consistent with the numerically obtained value.

In closing these considerations, let us point out the importance of the dashed line shown in Figure 10. For 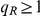, i.e., when RNA polymerase concentrations 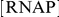 are as high as the dissociation constant 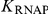 or higher and assuming the energy shifts 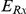 and 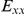 are negligibly small, then transcription can only exhibit a less-than-additive response to the activation by the transcription factors bound at the two promoter sites even if there is an arbitrarily strong interaction (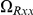 large) between the two transcription factor molecules and RNA polymerase. We are dealing here with a situation similar to the one reported in the Section ‘Nonlinearities of the thermostatistical transcriptional machinery’. In that section we found that under certain circumstances only less-than-additive effects can be observed when RNA polymerase concentrations are larger than half of the dissociation constant. These less-than-additive responses were predicted to hold irrespective of the precise values of the energy shifts 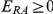 and 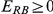 of RNA polymerase binding energy as induced by the individual transcription factors 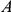 and 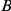.

#### Experimental case studies: illustration of synergistic activation for promoters with two activator binding sites

**Figure 12:**
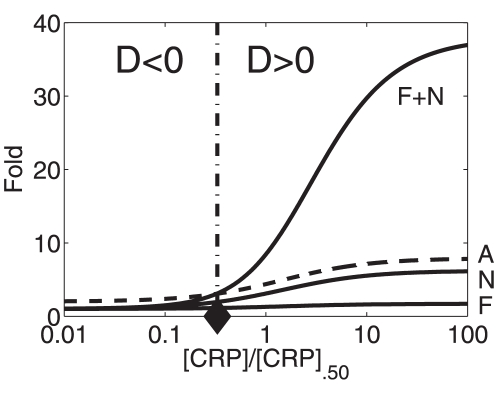
Dose responses, less-than-additive response domains, and greater-than-additive response domains predicted for the synthetic promoters studied by Joung et al. (1993). Solid lines labeled ‘F’ and ‘N’ were computed from Eq. (82) of Text S1 and represent predicted dose responses for the ‘far’ and ‘near’ single binding site promoters, respectively, of the Joung et al. study [5]. The dashed line indicates the sum of the activities of the single binding site promoters. The solid line labeled ‘F+N’ was computed from Eq. (25) and corresponds to the predicted dose response of the double binding site promoter. The diamond indicates the critical transcription factor dose obtained numerically from the intersection of the dashed graph with the ‘F+N’ graph. Parameters: see text.

As mentioned in the introduction, Joung et al. [5] and Busby et al. [14] engineered promoters with two binding sites (‘near’ and ‘far’) for the transcription factor CRP. In these studies it was found that the promoters with the two binding sites (‘near’ and ‘far’) induced a transcriptional activity than was higher than the sum of the activities recorded from the respective single-binding-site promoters (single ‘near’ site or single ‘far’ site promoters). This greater-than-additive effect is illustrated in Figure 1E–F. Following the procedure suggested in [8], we estimated the model parameters 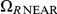, 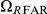, and 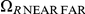 from the data reported by Joung et al. and Busby et al. We obtained: 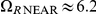, 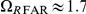, and 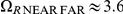 (Joung et al.) and 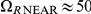, 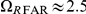, and 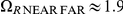 (Busby et al.). We found for both studies cooperative factors 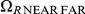 larger than unity which indicates that for such engineered promoters three-body interactions between RNA polymerase and the CRP transcription factors bound at the near and far promoter binding sites are relevant [8]. From the binding probability of the double-binding-site promoters defined by Eq. (25) and the analogous equations for the single-binding-site promoters (see Eq. (82) in Text S1), we computed the dose responses of the double-site and single-site promoters. This is illustrated in Figure 12 for the Joung et al. experiment. The dashed line is the sum of the predicted ‘far’ and ‘near’ single-site promoter activities. We found a cross-over from less-than-additive to greater-than-additive response at a critical value 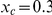 measured in units of 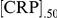 (this is the dose that would induce 50 percent of the saturation activity observed for the stronger, ‘near’ single-site promoter). For the Busby et al. experiment a critical value of 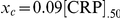 was found. In addition to expressing the critical values 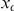 in units of 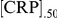, we also calculated them as relative concentrations. We obtained 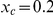 for the engineered promoters of Joung et al. and 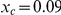 for the double-binding-site promoter investigated by Busby et al. (note: for the Busby et al. study we obtain 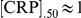). These two critical values differ by a factor of 2. Based on this observation, we may compare the domains in which the promoters of the Joung et al. and Busby et al. studies exhibit greater-than-additive responses. In doing so, we would conclude that the greater-than-additive domain of the Joung et al. double-site promoter is larger than the greater-than-additive domain of the Busby et al. double-site promoter.

## Discussion

### The reaction kinetics modeling and the thermostatistical modeling

We have derived a statistical model describing the combinatorial impact of multiple transcription factors on the RNAP binding probabilities and gene expression rates. We computed the probability of RNAP being bound to the promoter by calculating the sum of the probabilities of all DNA states 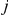 for which RNAP is bound at the promoter. The DNA state probabilities can be computed using a reaction kinetics approach or a thermostatistical approach (see Text S1 for details). The former method yields analytical expressions for concentrations 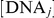 of DNA (or promoter) states 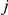. From Eq. (3) it follows that if the concentration 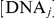 for a state 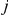 is larger (smaller) than the concentration 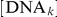 for state 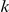, then the probability that the DNA is in state 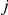 is larger (smaller) than the probability that the DNA is in state 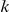:
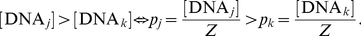
(28)The binding probabilities 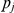 can be cast into the form (see Text S1)
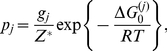
(29)where 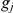 is the so-called degeneracy factor of statistical mechanics [45] computed in our context from ligand concentrations. From Eq. (29) it follows that if the standard free energy 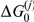 of a state 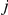 is lower (higher) than the standard free energy 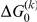 of a state 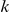 (when corrected for the degeneration factors) then the probability of observing the state 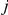 is larger (smaller) than the probability of state 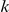. More precisely:
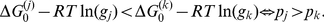
(30)In short, as a by-product of the derivation of our model for the regulation of transcriptional activity we showed that reaction kinetics approaches and thermostatistical free energy approaches yield consistent results.

### Implications for transcriptional activation by two activators

We focused on studying promoters regulated by two transcription factors. We found that in this case the binding probability 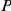 and the gene expression rate 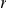 depend on 7 variables and parameters, which are the relative concentrations 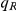, 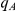, 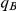 and the parameters 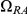, 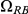, 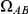, 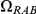. This implies that in order to examine the cooperative transcriptional activation of two transcription factors, we need in general to consider a 7-dimensional problem. In order to conduct a semi-analytical approach, we studied several special cases in more detail, see Tables 2 and 3. In doing so, we were able to examine semi-analytically differential characteristic conditions leading to less-than-additive and greater-than-addition effects. Moreover, we elaborated on how synergistic activation emerges when transcription factor concentrations are gradually increased.

First of all, we addressed the issue that in general, at low transcription factor concentrations only less-than-additive effects can be observed. Second, if three-body interactions between RNAP and the two transcription factors are negligibly small (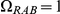) and if transcription factors do not interact among each other (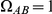), then critical boundary lines in the space spanned by 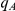, 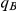, 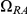, 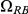 can be determined (see Eq. (21), Table 2, and Figure 2) that guarantee that only less-than-additive effects can be observed ‘below’ these critical boundaries. Likewise, we derived critical boundary values (see Eqs. (21), (23), Table 2, and Figures 2 and 4) such that only greater-than-additive effects can be observed ‘above’ these critical values. In this context, both less-than-additive and greater-than-additive effects are induced by the nonlinearities of the transcriptional machinery and do not result from three-body-interactions between RNAP and the two transcription factors. Most strikingly, we showed that if such three-body-interactions are negligibly small for a promoter under consideration then at relatively high RNAP concentrations greater-than-additive effects cannot be induced by any kind of transcription factor activity. The critical value obtained from our statistical model is 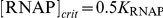, where 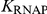 is the dissociation constant of RNAP. Consequently, we conclude that

- if a greater-than-additive effect disappears when the RNAP concentration is increased, then this can be taken as a hint that the greater-than-additive effect was caused by the nonlinearities of the transcriptional machinery and not by three-body-interactions between RNAP and the two transcription factors.

The critical concentration value 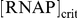 can alternatively be expressed in terms of the basal binding probability of RNAP at the promoter. From 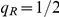 it follows that the critical basal binding probability 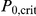 equals 1/3 (see Eq. (26) in Text S1). We conclude that

- if a synergistic greater-than-additive effect is observed for a promoter that exhibits a relatively high basal RNAP binding probability (i.e., 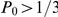) then this greater-than-additive effect is caused by three-body-interactions.

This is because the effect would be impossible in the absence of three-body interactions.

Indeed, the relative RNAP concentration 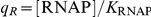 is to a certain extent accessible to experimental manipulations. For example, for several mutant 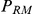 promoters of *E. coli* RNAP concentrations were varied from 0.01 to 0.1 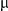
M and dissociation constants in the range of 0.001 to 0.01 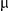
M were found [46]. In a related study on several different *E. coli* promoters, RNAP concentrations were scaled up from about 0.1 to 1 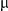
M. The relevant dissociation constants 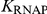 were found to be in a similar range as the RNAP concentrations, namely, in the range of 0.01 to 10 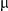
M [47]. A more recent study based on fluorescence anisotropy measurements reports from a dissociation constant 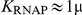
M for the lac promoter of *E. coli* and from RNAP concentrations that can be varied in a range of 0.1 to 1 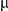
M [48]. In our context, this would imply that 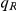 varies from 0.1 to 1. This interval includes the critical value of 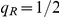. Using fluorescence anisotropy measurements again, in another study RNAP concentrations were varied in a considerably wide interval ranging from 0.01 to 100 nM. In this study 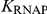 values for two promoters of *E. coli* were found to be of the order of 1 nM [49]. This implies that the basal binding probability 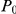 of RNAP scales effectively from 0 to 100 percent in the aforementioned 0.01 to 100 nM interval of RNAP concentrations. In this scenario, the critical value of 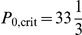 percent can be approached from both the lower and higher spectrum of basal binding probabilities.

Let us return to the observation that synergistic greater-than-additive effects can be induced merely by the nonlinearities of the transcriptional machinery [18]. According to our analysis, such nonlinearity-induced greater-than-additive effects are highly sensitive to the precise values of the RNA polymerase energy shifts induced by individual transcription factors. In this context, Figure 3 illustrates that ‘more’ does not necessarily mean ‘better’. If 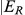 is too small or too large, greater-than-additive responses cannot be induced. We conclude that

- in the absence of significant three-body interactions transcription factors must be neither too weak nor too strong in order to induce a greater-than-additive transcriptional response due to cooperative stimulation.
- if the transcriptional machinery is re-entrant with respect to its binding-energy parameters then it is likely that the machinery must be fine-tuned in order to be able to produce greater-than-additive responses to cooperative activation.

Third, our analysis showed that three-body-interactions between RNAP and two transcription factors can indeed result in synergistic activation, i.e., a greater-than-additive effect. However, this is not necessarily the case in every circumstance. As illustrated in Figure 6, the transcriptional machinery may operate in a less-than-additive mode even if due to three-body-interactions (e.g., via looping or assembly of an activation complex) the chance of RNAP binding to the promoter is increased. More precisely, if the cooperative interaction parameter 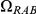 is smaller than 2, then depending on the magnitude of the energy shifts 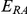 and 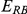 induced by the individual transcription factors, the promoter exhibits either a less-than-additive or a greater-than-additive response, see Figure 6. In view of these considerations, we conclude that

- three-body-interactions on the structural level and synergistic transcriptional effects on the gene expression level are as such two independent issues.

That is, looping or the assembly of an activation complex does not necessarily imply that the transcriptional machinery exhibits a synergy effect. Conversely, if a synergy effect cannot be observed this does not rule out the possibility that looping or the assembly of an activation complex is relevant for transcription. Note that the aforementioned critical value of 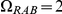 corresponds to an energy shift of 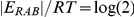. At room temperature this corresponds to a value of 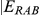 of about 750 J/mol or 0.2 kcal/mol. This critical value is smaller in the amount than the energy shift 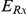 of about 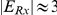 kcal/mol that a single transcription factor 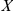 induces on RNAP as reported recently [50]. Likewise, interaction energies 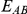 between two transcription factors 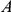 and 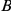 have been reported to be typically somewhat larger in magnitude, namely, 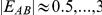 kcal/mol [51]. Note however that the energy shifts 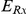 and 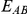 refer to interactions different from the three-body interactions yielding to energy shifts 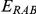. Finally, recall that the crude estimate for 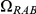 reported above in the context of the study by Lee et al. was of the order of the critical value 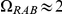.

### Dose responses

At the beginning of the previous section on the impact of two activators, we elaborated on how cooperative activation exhibiting a greater-than-additive response as a phenomenon emerges when transcription factor concentrations are increased. We demonstrated that less-than-additive transcriptional responses at low transcription factor concentrations will turn into greater-than-additive responses when transcription factor concentrations exceed certain critical values. We derived critical values both for greater-than-additive effects caused by the nonlinearities of the transcriptional machinery (see Text S1, Eq. (91)) and induced by three-body-interactions (see Eq. (26)). However, these critical values hold for the special case in which the promoter exhibits two binding sites for one transcription factor. The general case of two different transcription factors acting on the promoter can be addressed using the analytical expression of the binding probability 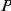 defined on the abovementioned 7-dimensional space, see Eq. (15). In general, this has to be done numerically.

**Figure 13:**
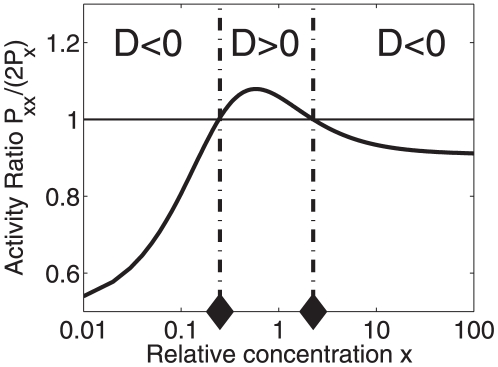
Illustration of a re-entrant dose response predicted by the thermostatistical model for a double-binding-site promoter using the measure of synergy suggested by Chi and Carey (1996). The graph represents the measure of synergy 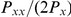 proposed by Chi and Carey [15] calculated from the ‘DS’ and ‘2× SS’ graphs shown in Figure 9. After reaching a peak value the graph decays monotonically as a function of 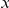 and leaves the greater-than-additive response domain at a certain critical transcription factor concentration. Such a behavior was observed in experiments by Chi and Carey on a promoter with seven binding sites, see Figure 1G–H.

Our analysis suggests that in general the dose response to a combined stimulation by means of two transcription factors can exhibit a re-entrant pattern. We investigated such re-entrant patterns explicitly for promoters with two binding sites for the same transcription factor. To this end, we used the difference measure 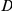, which is positive for greater-than-additive responses and negative for less-than-additive responses. Accordingly, re-entrant gene expression levels induced by monotonically increasing transcription factor concentrations correspond to sequences 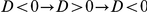, see Figure 9. An alternative measure –– more closely related to the experimental study by Chi and Carey [15] –– is the ratio of activity induced by a promoter with two binding sites relative to two times the activity of a modified version of the promoter with a single binding site only. Mathematically speaking, this ratio is given by 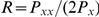 with 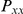 defined by Eq. (25) and 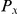 given by Eq. (82) in Text S1. For this measure less-than-additive and greater-than-additive responses are defined by 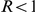 and 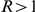, respectively. In particular, re-entrant dose responses correspond to sequences 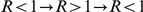, see Figure 13. From Figure 13 it is clear that transcriptional activity of promoters operating in this re-entrant mode decays monotonically for large enough stimulations (i.e., transcription factor concentrations 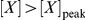, where 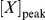 is the concentration that induces the peak transcriptional activity). As mentioned in the introduction and illustrated in Figure 1G–H, Chi and Carey observed such a monotonically decaying activity pattern [15]. Moreover, an increasing and finally decreasing dose response was also suggested in a related study [16] (see the discussion of Fig. 5 in [16]). Using a thermostatistical approach similar to the one developed above, Wang et al. [22] fitted the gene expression data to a single-peaked response function that qualitatively corresponds to the graph shown in Figure 13. As opposed to the modeling study by Wang et al., which was purely computational, our rigorous mathematical analysis yields the critical values for the re-entrant phenomenon, and in doing so gives a clear proof to the existence of re-entrant dose response patterns. Note also that Chi and Carey studied a promoter involving seven binding sites for the transcription factor and considered the situation in which an activation complex is assembled due to the synergistic impact of transcription factor molecules bound at those seven promoter sites. In contrast, we showed that the single-peaked response function is predicted even for promoters involving only two binding sites and can arise merely from the nonlinearities of the thermostatistical transcriptional machinery.

Moreover, our analysis suggests that the monotonically decaying dose response observed in the study by Chi and Carey actually belongs to a family of three possible response patterns, which are summarized in Table 3. We speculate that under appropriate experimental conditions (e.g., when the impact of the activators is manipulated [14]) one could observe also one of the two alternative, qualitatively different dose-response patterns.

Finally, as argued in the introduction, the re-entrant case implies that the transcriptional machinery under consideration requires at least some degree of fine-tuning. Perturbations in transcription factor concentrations may shift the transcriptional machinery out of the operational domain in which cooperative activation produces a greater-than-additive effect. Importantly, in the context of the re-entrant case, perturbations in both directions (i.e., yielding higher or lower transcription factor doses) can induce a change from a greater-than-additive response to a less-than-additive response.

## Supporting Information

Text S1 **Provides the following information: In Section 1 a rigorous mathematical derivation of the thermostatistical model (7) for multiple transcription factors is given.** In Section 2 the model is compared with the thermostatistical model for transcription initiation proposed by Shea and Ackers. Section 3 provides mathematical details of the thermostatistical model for two activators. In Section 4 a proof is given that the thermostatistical modeling approach predicts that gene expression regulated by two activators is a monotonically increasing function of the activator concentrations. Section 5 provides various mathematical proofs necessary to show under which conditions less-than-additive and greater-than-additive effects are predicted by the thermostatistical two activator model for transcription initiation.
(PDF)
