# Supplementary material for: Versatility of Cooperative Transcriptional Activation: A Thermodynamical Modeling Analysis for Greater-Than-Additive and Less-Than-Additive Effects
Source: PLoS One. 2012 Apr 10;7(4):e34439. doi: 10.1371/journal.pone.0034439 (PMC3323628; doi:10.1371/journal.pone.0034439)
Supplement: Text S1 — Provides the following information: In Section 1 a rigorous mathematical derivation of the thermostatistical model (7) for multiple transcription factors is given. In Section 2 the model is compared with the thermostatistical model for transcription initiation proposed by Shea and Ackers. Section 3 provides mathematical details of the thermostatistical model for two activators. In Section 4 a proof is given that the thermostatistical modeling approach predicts that gene expression regulated by two activators is a monotonically increasing function of the activator concentrations. Section 5 provides various mathematical proofs necessary to show under which conditions less-than-additive and greater-than-additive effects are predicted by the thermostatistical two activator model for transcription initiation. (PDF) [file pone.0034439.s001.pdf]

SUPPORTING TEXT S1

Versatility of Cooperative Transcriptional Activation: a Thermodynamical Modeling Analysis for Greater-Than-Additive and Less-Than-Additive Effects

T. D. Frank[1,2,*], A. M. Carmody[1], B. N. Kholodenko[1]

1 Systems Biology Ireland, University College Dublin, Belfield, Dublin 4, Ireland
2 UCD School of Physics, University College Dublin, Belfield, Dublin 4, Ireland
∗ E-mail: till.frank@ucd.ie

Contents

1 Derivation of the general thermostatistical model: multiple transcription factors 1

2 Multiple transcription factor model in the formulation by Shea and Ackers 4

3 Two activator model and the regulating function 4

4 Monotonical regulation of gene expression by two activators 5

5 Less-then-additive and greater-than-additive effects 7
- 5.1 Difference measure for two activators . . . 7
  - 5.1.1 Low transcription factor concentrations . . . 7
  - 5.1.2 Weak activators . . . 7
  - 5.1.3 Nonlinearities of the thermostatistical transcriptional machinery . . . 8
  - 5.1.4 Three-body interactions . . . 11
  - 5.1.5 Saturation case at low RNAP concentrations . . . 11
- 5.2 Cross-over behavior induced by the dose increase of transcription factors . . . 12
  - 5.2.1 Cross-over responses and re-entrant responses caused by nonlinearities of the thermostatistical transcriptional machinery . . . 13
  - 5.2.2 Cross-over dose responses induced by three-body interactions . . . 14

1 Derivation of the general thermostatistical model: multiple transcription factors

As mentioned in the main text, DNA states j can conveniently be described by means of chemical reaction equations of the form

$$\mathrm{DNA}_{j=1} + m_j\,\mathrm{RNAP} + \sum_{n=1}^{N} m_{jn}\,T_n \leftrightarrow \mathrm{DNA}_j \tag{1}$$

for $j = 1, \ldots, L$, where $(m_1, \ldots, m_L)$ is a vector with $m_j \in \{0, 1\}$, m_{jn} are the stoichiometric coefficients of a $L \times N$ matrix, and T_n are the aforementioned transcription factors $n = 1, \ldots, N$.

Note that $m_j = 1$ ($m_j = 0$) indicates that in the state j RNA polymerase is (is not) bound to the promoter.

Let us determine the probability p_j to find a cell in a particular DNA state j when selecting randomly a cell out of a population of cells. To this end, we determine from each reaction the equilibrium concentration of DNA in state j and the probabilities p_j that these states can be observed.

We first consider the change of the molar free energy $\Delta G^{(j)}$ when the reaction described by Eq. (1) is not in chemical equilibrium [1]

$$\Delta G^{(j)} = G_{\mathrm{DNA}\,j} - G_{\mathrm{DNA}\,j=1} - m_j\, G_{\mathrm{RNAP}} - \sum_{n=1}^{N} m_{jn}\, G_n \ . \tag{2}$$

Here $G_{\mathrm{DNA}\,j}$ and G_{RNAP} denote the molar free energies of the DNA state j and the RNA polymerase, respectively, and G_n denote the molar free energies of the transcription factors $n = 1, \ldots, N$. Let $G_{\mathrm{DNA}\,j,0}$ and $G_{\mathrm{RNAP},0}$, and $G_{n,0}$ denote the corresponding standard free energies (for standard unit concentrations). Substituting $G_X = G_{X,0} + RT \log[X]$ [1] (where T is temperature, R is the Boltzmann gas constant, and $[X]$ is the concentration of X) into Eq. (2), we obtain

$$\Delta G^{(j)} = \Delta G_0^{(j)} + RT \ln \left(\frac{[\mathrm{DNA}_j]}{[\mathrm{DNA}_{j=1}][\mathrm{RNAP}]^{m_j} \prod_{n=1}^{N} [T_n]^{m_{jn}}} \right) \tag{3}$$

with

$$\Delta G_0^{(j)} = G_{\mathrm{DNA}\,j,0} - G_{\mathrm{DNA}\,j=1,0} - m_j\, G_{\mathrm{RNAP},0} - \sum_{n=1}^{N} m_{jn}\, G_{n,0} \ . \tag{4}$$

In chemical equilibrium, the concentrations of the reactants occurring in Eq. (1) satisfy

$$[\mathrm{DNA}_j] = \frac{[\mathrm{DNA}_{j=1}][RNAP]^{m_j} \prod_{n=1}^{N} [T_n]^{m_{jn}}}{K_D^{(j)}} \ , \tag{5}$$

where $K_D^{(j)}$ is the dissociation constant of the reaction. Likewise, in chemical equilibrium we have $\Delta G^{(j)} = 0$ (which implies $\Delta G_0^{(j)} = RT \log K_D^{(j)}$), and consequently from Eqs. (3,4) we obtain

$$G_{\mathrm{DNA}\,j,0} - G_{\mathrm{DNA}\,j=1,0} - m_j\, G_{\mathrm{RNAP},0} - \sum_{n=1}^{N} m_{jn}\, G_{n,0} = -RT \ln \left(\frac{[\mathrm{DNA}_j]}{[\mathrm{DNA}_{j=1}][\mathrm{RNAP}]^{m_j} \prod_{n=1}^{N} [T_n]^{m_{jn}}} \right) \ . \tag{6}$$

Let us discuss the free energy difference $G_{\mathrm{DNA}\,j,0} - G_{\mathrm{DNA}\,j=1,0}$. When considering concentrations of one molar, what are the contributions that affect the DNA free energy when we compare state $j = 1$ with state $j \neq 1$?

First of all, the DNA state j includes the free energies of the bound RNAP and the bound transcription factors. Let us denote these standard free energies by $G_{\mathrm{RNAP},0,\mathrm{bound}}$ and $G_{n,0,\mathrm{bound}}$ in order to distinguish them from the standard free energies of the corresponding unbounded ligands $G_{\mathrm{RNAP},0}$ and $G_{n,0}$.

Second, changes of the free energy are due to various impacts of transcription factors. Activators lower the binding energy of RNAP by a certain amount. We will denote such energy shifts by $E_{Rn} \leq 0$. Two transcription factors may affect the RNAP binding energy by mechanisms that cannot be induced by single transcription factors alone. Energy shifts induced by such mechanisms will be denoted by $E_{Rni} \leq 0$. Higher order interactions can be described in terms of E_{Rnik} and so on. In addition, interactions between transcription factors that do not involve RNA polymerase may affect the free energy. We account for such interactions by introducing energy shift terms denoted by E_{ni}. Again, higher order interactions may be described in terms of expressions like E_{nik}. In summary, we put

$$G_{\mathrm{DNA}\,j,0} \quad = \quad G_{\mathrm{DNA}\,j=1,0} + m_j\, G_{\mathrm{RNAP},0,\mathrm{bound}} + \sum_{n=1}^{N} m_{jn}\, G_{n,0,\mathrm{bound}}$$

$$+m_j\left(\sum_{n=1}^{N} m_{jn}\,E_{Rn} + \sum_{n,i\in I^{(j)}} E_{Rni}\right) + \sum_{n,i\in I^{(j)}} E_{ni}$$
$$+\text{higher-order interactions}\ , \tag{7}$$

where the index-sets $I^{(j)}$ are defined by all transcription factors that are involved in the state j: $I^{(j)} = \{k = 1, \ldots, N \mid m_{jk} > 0\}$. Aiming at a more concise description, we introduce (in analogy to the aforementioned relationship $\Delta G_0^{(j)} = RT \log K_D^{(j)}$) the dissociation constants K_{RNAP} and K_n as follows:

$$\begin{aligned} G_{\text{RNAP},0,\text{bound}} - G_{\text{RNAP},0} &= RT \ln K_{\text{RNAP}}\ , \\ G_{n,0,\text{bound}} - G_{n,0} &= RT \ln K_n\ . \end{aligned} \tag{8}$$

Likewise, we define the parameters Ω_{Rn} and cooperativity factors Ω_{Rni} and Ω_{ni} like

$$\begin{aligned} -RT \ln \Omega_{Rn} &= E_{Rn}\ , \\ -RT \ln \Omega_{Rni} &= E_{Rni}\ , \\ -RT \ln \Omega_{ni} &= E_{ni} \end{aligned} \tag{9}$$

with $\Omega > 0$ for all Ω-parameters. If all transcription factors correspond to activators then the energy shifts listed in Eq. (9) are assumed to be negative which implies that the Ω-parameters are larger than 1: $\Omega > 1$. Neglecting the higher-order interactions indicated in Eq. (7), we obtain from Eqs. (6,...,9)

$$[\text{DNA}_j] = [\text{DNA}_1]\, q_R^{m_j} \prod_{n=1}^{N} q_n^{m_{jn}} \left(\prod_{n=1}^{N} \Omega_{Rn}^{m_{jn}} \prod_{n,i\in I^{(j)}} \Omega_{Rni}\right)^{m_j} \prod_{n,i\in I^{(j)}} \Omega_{ni}\ , \tag{10}$$

where we have used the dimensionless, relative concentrations (see main text)

$$q_R = \frac{[\text{RNAP}]}{K_{\text{RNAP}}}\ ,\ q_n = \frac{[T_n]}{K_n}\ . \tag{11}$$

Note that Eq. (10) describes equilibrium concentrations only, whereas Eq. (3) holds both in chemical equilibrium and non-equilibrium.
Having derived an expression for $[\text{DNA}_j]$, we define next the probability of observing a randomly selected cell in a DNA state j by [2]

$$p_j = \frac{[\text{DNA}_j]}{Z} \tag{12}$$

with the partition function $Z = \sum_{j=1}^{L} [\text{DNA}_j]$. We see that the expression $[\text{DNA}_{j=1}]$ can be dropped out of the equation by defining the fractions as follows,

$$[\text{DNA}_j]^* = \frac{[\text{DNA}_j]}{[\text{DNA}_1]} = q_R^{m_j} \prod_{n=1}^{N} q_n^{m_{jn}} \left(\prod_{n=1}^{N} \Omega_{Rn}^{m_{jn}} \prod_{n,i\in I^{(j)}} \Omega_{Rni}\right)^{m_j} \prod_{n,i\in I^{(j)}} \Omega_{ni}\ , \tag{13}$$

which can be used to calculate p_j like

$$p_j = \frac{[\text{DNA}_j]^*}{Z^*} \tag{14}$$

with $Z^* = \sum_{j=1}^{L} [\text{DNA}_j]^*$. Note that by substituting the energy shifts and interaction energies (9) into Eqs. (13,14) the probability p_j can equivalently be expressed by

$$p_j = \frac{1}{Z^*}\, q_R^{m_j} \prod_{n=1}^{N} q_n^{m_{jn}}\ \exp\left\{ -\frac{m_j\left(\sum_{n=1}^{N} m_{jn}\,E_{Rn} + \sum_{n,i\in I^{(j)}} E_{Rni}\right) + \sum_{n,i\in I^{(j)}} E_{ni}}{RT} \right\}\ . \tag{15}$$

It can be shown that this formulation is consistent with the formulation proposed by Shea and Ackers (see below).

2 Multiple transcription factor model in the formulation by Shea and Ackers

Above (see Eqs. (4), (7), and (15)) we derived the equations

$$\Delta G_0^{(j)} = G_{\mathrm{DNA}\,j,0} - G_{\mathrm{DNA}\,j=1,0} - m_j\, G_{\mathrm{RNAP},0} - \sum_{n=1}^{N} m_{jn}\, G_{n,0} \;, \tag{16}$$

$$\begin{aligned} G_{\mathrm{DNA}\,j,0} \;&=\; G_{\mathrm{DNA}\,j=1,0} + m_j\, G_{\mathrm{RNAP},0,\mathrm{bound}} + \sum_{n=1}^{N} m_{jn}\, G_{n,0,\mathrm{bound}} \\ &\quad + m_j \left(\sum_{n=1}^{N} m_{jn}\, E_{Rn} + \sum_{n,i \in I^{(j)}} E_{Rni} \right) + \sum_{n,i \in I^{(j)}} E_{ni} \;, \end{aligned} \tag{17}$$

and

$$p_j = \frac{1}{Z^*}\, q_R^{m_j} \prod_{n=1}^{N} q_n^{m_{jn}}\; \exp\left\{ -\frac{m_j \left(\sum_{n=1}^{N} m_{jn}\, E_{Rn} + \sum_{n,i\in I^{(j)}} E_{Rni} \right) + \sum_{n,i\in I^{(j)}} E_{ni}}{RT} \right\} . \tag{18}$$

Substituting Eq. (17) into Eq. (16), we obtain

$$\begin{aligned} \Delta G_0^{(j)} \;&=\; m_j [G_{\mathrm{RNAP},0,\mathrm{bound}} - G_{\mathrm{RNAP},0}] + \sum_{n=1}^{N} m_{jn} [G_{n,0,\mathrm{bound}} - G_{n,0}] \\ &\quad + m_j \left(\sum_{n=1}^{N} m_{jn}\, E_{Rn} + \sum_{n,i \in I^{(j)}} E_{Rni} \right) + \sum_{n,i \in I^{(j)}} E_{ni} \;. \end{aligned} \tag{19}$$

Substituting this result into Eq. (18) with the help of Eqs. (8,11), we obtain

$$p_j = \frac{1}{Z^*}\, [\mathrm{RNAP}]^{m_j} \prod_{n=1}^{N} [T_n]^{m_{jn}}\; \exp\left\{ -\frac{\Delta G_0^{(j)}}{RT} \right\} \;, \tag{20}$$

which corresponds to the formulation of multiple transcription factor models proposed by Shea and Ackers [3, 4, 5].

3 Two activator model and the regulating function

In the main text (see Eq. (15)) we derived the thermostatistical model for two activators:

$$P = \frac{q_R\, (1 + q_A\, \Omega_{RA} + q_B\, \Omega_{RB} + q_A\, q_B\, \Omega_{RA}\, \Omega_{RB}\, \Omega_{AB}\, \Omega_{RAB})}{q_R\, (1 + q_A\, \Omega_{RA} + q_B\, \Omega_{RB} + q_A\, q_B\, \Omega_{RA}\, \Omega_{RB}\, \Omega_{AB}\, \Omega_{RAB}) + 1 + q_A + q_B + q_A\, q_B\, \Omega_{AB}} \;. \tag{21}$$

Eq. (21) can alternatively be expressed by

$$P = \frac{1}{1 + \mathrm{RNAP}_{\mathrm{off}}/\mathrm{RNAP}_{\mathrm{on}}} \;. \tag{22}$$

Introducing the function F_{reg} like

$$q_R\, F_{\mathrm{reg}} = \frac{\mathrm{RNAP}_{\mathrm{on}}}{\mathrm{RNAP}_{\mathrm{off}}} \;. \tag{23}$$

we obtain for $F_{\rm reg}$ the expression

$$F_{\rm reg} = \frac{1 + q_A\,\Omega_{RA} + q_B\,\Omega_{RB} + q_A\,q_B\,\Omega_{RA}\,\Omega_{RB}\,\Omega_{AB}\,\Omega_{RAB}}{1 + q_A + q_B + q_A\,q_B\,\Omega_{AB}} \ . \tag{24}$$

and the probability P of observing RNA polymerase being bound at the promoter becomes

$$P = \frac{q_R}{q_R + 1/F_{\rm reg}} \ . \tag{25}$$

Importantly, in the absence of any transcription factor we have $F_{\rm reg} = 1$ and P described the basal binding probability of RNA polymerase at the promoter. In the presence of transcription factors $F_{\rm reg}$ becomes different from unity, which affects the binding probability of RNA polymerase. Therefore, it has been suggested to refer to $F_{\rm reg}$ as regulatory function (whence the subindex 'reg') for transcription initiation [6, 7].

4 Monotonical regulation of gene expression by two activators

In this section, we consider several limiting cases and in particular show that the binding probability P increases monotonically as function of the activator concentrations $[T_A]$ and $[T_B]$. First of all, the basal binding probability P_0 related to the basal transcription rate $r_0 = \beta P_0$ is defined by (see Eq. 21 or Eq. 25 with $F_{\rm reg} = 1$)

$$P_0 = P(q_A = 0, q_B = 0) = \frac{q_R}{1 + q_R} \ . \tag{26}$$

Next, let us study how binding probability P is affected by the impacts of individual transcription factors. Let $P(q_A) = P(q_A, q_B = 0)$ denote the binding probability of RNA polymerase at the promoter under the impact of the transcription factor A in the absence of the transcription factor B. From Eq. (21) it follows that

$$P(q_A) = \frac{q_R(1 + q_A\,\Omega_{RA})}{q_R(1 + q_A\,\Omega_{RA}) + 1 + q_A} \ . \tag{27}$$

We re-obtain the basal binding probability for

$$P(q_A = 0) = P_0 \tag{28}$$

and define the saturation probability $P_{A,sat}$ like

$$P_{A,sat} = P(q_A \to \infty) = \frac{q_R\,\Omega_{RA}}{1 + q_R\,\Omega_{RA}} \approx P(q_A \gg 1) \ . \tag{29}$$

Note that the case $q_A \gg 1$ implies that $\Omega_{RA}\,q_A \gg 1$ holds because we have $\Omega_{RA} \geq 1$ for activators. From $\Omega_{RA}\,q_A \gg 1$ it follows that we can neglect the additive '+1' term such that $1 + \Omega_{RA}\,q_A \approx \Omega_{RA}\,q_A$. This is an approximation as indicated in Eq. (29). The question arises whether the saturation probability $P_{A,sat}$ is higher than the basal binding probability P_0 (which would be consistent with our notion of an activator). In order to answer this question note that the function

$$f(z) = \frac{z}{1 + z} \tag{30}$$

(which is just the Michaelis-Menten enzyme kinetics saturation curve) is known to increase monotonically from $f(z = 0) = 0$ to $f(z \to \infty) = 1$. Since we have $P_0 = f(z = q_R)$ and $P_{A,sat} = f(z = q_R\,\Omega_{RA})$ and $\Omega_{RA} \geq 1$ we conclude that $P_{A,sat} \geq P_0$. In particular, for $\Omega_{RA} > 1$ we have

$P_{A,sat} > P_0$. Moreover, differentiating $P(q_A)$ with respect to q_A and taking again $\Omega_{RA} \geq 1$ into account gives us

$$\frac{\mathrm{d}}{\mathrm{d}q_A} P(q_A) = q_R \frac{\Omega_{RA} - 1}{[1 + q_R + q_A(1 + q_R\,\Omega_{RA})]^2} \geq 0 \tag{31}$$

and $\mathrm{d}P/\mathrm{d}q_A > 0$ for $\Omega_{RA} > 1$. In short, the binding probability and consequently the transcription rate r increases monotonically as a function of activator concentration $[T_A]$ assuming that the activator decreases the binding energy of RNA polymerase (i.e., assuming that $E_{RA} < 0$ or $\Omega_{RA} > 1$). By analogy, for the transcription factor B we obtain

$$P(q_B) = P(q_A = 0, q_B) = \frac{q_R(1 + q_B\,\Omega_{RB})}{q_R(1 + q_B\,\Omega_{RB}) + 1 + q_B} \tag{32}$$

with

$$P(q_B = 0) = P_0 \tag{33}$$

and

$$P_{B,sat} = P(q_B \to \infty) = \frac{q_R\,\Omega_{RB}}{1 + q_R\,\Omega_{RB}} \approx P(q_B \gg 1) \tag{34}$$

with $P_{B,sat} \geq P_0$. The function $P(q_B)$ increases monotonically with $[T_B]$ for negative energy shifts $E_{RB} < 0$ $(\Omega_{RB} > 1)$.
In the presence of both activators A and B the saturation binding probability is defined by

$$P_{AB,sat} = P(q_A \to \infty, q_B \to \infty) \approx P(q_A \gg 1, q_B \gg 1)\ . \tag{35}$$

In this limiting case the quadratic terms in Eq. (21) dominate the linear and constant terms. Consequently, we obtain

$$P_{AB,sat} = \frac{q_R\,\Omega_{RA}\,\Omega_{RB}\,\Omega_{AB}\,\Omega_{RAB}}{1 + q_R\,\Omega_{RA}\,\Omega_{RB}\,\Omega_{AB}\,\Omega_{RAB}} = f(q_R\,\Omega_{RA}\,\Omega_{RB}\,\Omega_{AB}\,\Omega_{RAB}) \geq P_0\ . \tag{36}$$

For $\Omega_{RA}\,\Omega_{RB}\,\Omega_{AB}\,\Omega_{RAB} > 1$ we have $P_{AB,sat} > P_0$.
In order to show that the probability $P(q_A, q_B)$ increases monotonically in both directions q_A and q_B we compute the partial derivative $\partial P(q_A, q_B)/\partial q_A$ for fixed q_B. For sake of readability, we put $q_A = x$. Then, Eq. (21) can equivalently be expressed as

$$P(x, q_B) = \frac{xe + g}{x(e + w) + g + h} \tag{37}$$

with

$$\begin{aligned} e &= q_R\,\Omega_{RA}(1 + q_B\,\Omega_{RB}\,\Omega_{AB}\,\Omega_{RAB})\ , \\ w &= 1 + q_B\Omega_{AB}\ , \\ g &= q_R(1 + q_B\Omega_{RB})\ , \\ h &= 1 + q_B\ . \end{aligned} \tag{38}$$

First we check whether or not $P(x \to \infty, q_B) \geq P(x = 0, q_B)$ holds for all q_B. We have

$$P(x = 0, q_B) = \frac{g}{g + h}\ ,\quad P(x \to \infty, q_B) = \frac{e}{e + w}\ . \tag{39}$$

We see that $I = P(x \to \infty, q_B) - P(x = 0, q_B) = I^*/[(e + w)(g + h)]$ with

$$I^* = eh - gw\ . \tag{40}$$

A detailed calculation shows that

$$\frac{I^*}{q_R} = R + Uq_B + Vq_B^2 \tag{41}$$

with

$$
\begin{aligned}
R &= \Omega_{RA} - 1 \geq 0 \ , && (42)\\
U &= \Omega_{RA} + \Omega_{RA}\,\Omega_{RB}\,\Omega_{AB}\,\Omega_{RAB} - \Omega_{RB} - \Omega_{AB} \ , && (43)\\
V &= \Omega_{RB}\,\Omega_{AB}(\Omega_{RA}\,\Omega_{RAB} - 1) \geq 0 \ . && (44)
\end{aligned}
$$

In order to determine the sign of the expression U we consider first the special case $\Omega_{AB} = \Omega_{RAB} = 1$. In this case we have $U = (\Omega_{RA} - 1)(\Omega_{RB} + 1) \geq 1$ because we have $\Omega_{RA}, \Omega_{RB} \geq 1$. From Eq. (43) it is clear that U increases monotonically as function of Ω_{AB} and Ω_{RAB}. Consequently, we have $U \geq 0$ for all $\Omega_{RA}, \Omega_{RB}, \Omega_{AB}, \Omega_{RAB} \geq 1$. This implies that $I^* \geq 0$ and $I \geq 0$ for all q_B. Consequently, we have shown that $P(x \to \infty, q_B) \geq P(x = 0, q_B)$ holds irrespective of q_B. Having obtained this intermediate result, we can compute the partial derivative $\partial P(x, q_B)/\partial x$. From Eq. (37) we obtain

$$\frac{\partial}{\partial x} P(x, q_B) = \frac{I^*}{[x(e + w) + g + h]^2} \geq 0 \tag{45}$$

with I^* defined in Eq. (40). By analogy, we find

$$\frac{\partial}{\partial y} P(q_A, y) \geq 0 \tag{46}$$

for $y = q_B$. In summary, the function $P(q_A, q_B)$ is a monotonically increasing function with respect to the relative concentrations q_A and q_B provided that we assume that the energy shifts E_{RA}, E_{RB}, E_{RAB} induced by the transcription factors A and B as well the two-body interaction energy E_{AB} are negative.

5 Less-then-additive and greater-than-additive effects

5.1 Difference measure for two activators

In the main text we introduced the difference

$$D = P(q_A, q_B) - [P(q_A) + P(q_B)] \tag{47}$$

that actually is a function of the seven dimensional vector

$$\mathbf{x} = (q_R, q_A, q_B, \Omega_{RA}, \Omega_{RB}, \Omega_{AB}, \Omega_{RAB}) \ . \tag{48}$$

If $D > 0$ ($D < 0$) we have a greater-than-additive (less-than-additive) effect. In the following sections, we conduct a rigorous mathematical analysis in order to determine relevant subspaces of the seven dimensional space spanned by $\mathbf{x}$ in which either $D < 0$ or $D > 0$ holds. That is, we will discuss some of the proofs leading to the results summarized in Tables 2 and 3 of the main text.

5.1.1 Low transcription factor concentrations

At $q_A = q_B = 0$ we have

$$D = -P_0 = -\frac{q_R}{1 + q_R} < 0 \ , \tag{49}$$

see Eqs. (26), (28), (33). Since $P(q_A, q_B)$ and $D(q_A, q_B)$ are continuous functions in q_A and q_B, for sufficiently low concentrations $q_A \approx 0$ and $q_B \approx 0$ the quantity D remains negative. Mathematically speaking, Eq. (49) implies that there exists a parameter $\epsilon > 0$ such that

$$D < 0 \ \forall \ 0 \leq q_A^2 + q_B^2 < \epsilon \ . \tag{50}$$

We will derive below several cases in which critical values for ϵ can be derived, see Eq. (60) and Section 5.2 ('Cross-over behavior induced by the dose increase of transcription factors').

5.1.2 Weak activators

According to the model defined by Eq. (21), the transcription factors A and B have an impact on the transcription rate r and the binding probability P only if they interact with RNAP either independently via energy shifts E_{RA} and E_{RB} or via the three-body interaction E_{RAB}. If the transcriptional machinery only exhibits an interaction between the transcription factors A and B then it is intuitively clear that the binding probability P and consequently the transcription rate r is not affected by the transcription factors. In fact, if we put $\Omega_{RA} = \Omega_{RB} = \Omega_{RAB} = 1$ (i.e., use $E_{RA} = E_{RB} = E_{RAB} = 0$) then Eq. (21) reads

$$P(q_A, q_B) = \frac{q_R\, s}{q_R\, s + s} = \frac{q_R}{1 + q_R} = P_0 \tag{51}$$

with $s = 1 + q_A + q_B + q_A\, q_B\, \Omega_{AB}$. Likewise, from Eqs. (27) and (32) we obtain $P(q_A) = P_0\ \forall\ q_A$ and $P(q_B) = P_0\ \forall\ q_B$ such that

$$D = -P_0 < 0\ \forall\ q_A,\, q_B\ . \tag{52}$$

Since $P(q_A, q_B)$ and $D(q_A, q_B)$ are continuous functions in Ω_{RA}, Ω_{RB}, and Ω_{RAB} the quantity D remains negative for sufficiently weak activators, i.e., for activators with $\Omega_{RA}, \Omega_{RB}, \Omega_{RAB} \approx 1$ (or $E_{RA}, E_{RB}, E_{RAB} \approx 0$).

5.1.3 Nonlinearities of the thermostatistical transcriptional machinery

We study the impact of the energy shifts E_{RA} and E_{RB} on the binding probability P. To this end, we put $E_{AB} = E_{RAB} = 0$ (i.e., $\Omega_{AB} = \Omega_{RAB} = 1$). Note that the energy shifts E_{RA} and E_{RB} are induced independently by the transcription factors. Nevertheless for $\Omega_{AB} = \Omega_{RAB} = 1$ the RNA polymerase binding probability P defined by Eq. (21) is a nonlinear function with respect to q_A and q_B. Due to this nonlinearity the transcriptional machinery may exhibit greater-than-additive and less-than-additive effects.

For sake of readability, we put $q_A = x$ and $q_B = y$. Substituting Eq. (21), (27) and (32) into Eq. (47) we obtain

$$D(x, y) = \frac{\tilde{D}(x, y)}{Z_{xy} Z_x Z_y} \tag{53}$$

with the normalization factors given by

$$\begin{aligned} Z_{xy} &= q_R(1 + x\,\Omega_{RA})(1 + y\,\Omega_{RB}) + (1 + x)(1 + y) > 0\ , \\ Z_x &= q_R(1 + x\,\Omega_{RA}) + 1 + x > 0\ , \\ Z_y &= q_R(1 + y\,\Omega_{RB}) + 1 + y > 0\ . \end{aligned} \tag{54}$$

The quantity of interest is the nominator $\tilde{D}$ that determines the sign of D. A detailed calculation shows

$$\tilde{D}(x, y) = \underbrace{q_R(1 - 2q_R)(1 + x)(1 + y)uv}_{\text{'first term'}} - q_R\left[(1 + x)v + (1 + y)u\right](1 + x)(1 + y) - q_R^3 u^2 v^2 \tag{55}$$

with

$$u(x) = 1 + x\Omega_{RA}\ , \quad v(y) = 1 + y\Omega_{RB}\ . \tag{56}$$

The first term on the right-hand-side of Eq. (55) can be positive or negative. The second and third terms occurring in Eq. (55) are negative. Consequently, greater-than-additive effects due to nonlinearities must come from that first expression. More precisely, the first term must outbalance the second and third terms.

We see immediately that if RNAP concentrations are greater than half of the RNAP dissociation constant (i.e., $q_R \geq 1/2$), then the first term is negative and so is $\tilde{D}$. Consequently, we have

$$q_R \geq 0.5 \Rightarrow D < 0\ \forall q_A, q_B\ . \tag{57}$$

That is, nonlinearities cannot contribute to greater-than-additive effects at relative high RNA polymerase concentrations.
Let us next consider the case $q_R < 0.5$. For $q_A = q_B = 0$ we obtain $\tilde{D} = -q_R(1+q_R)^2 < 0$ which is consistent with the result derived in Section 5.1.1 ('Low transcription factor concentrations'). For $q_A, q_B > 0$ we break down Eq. (55) into components like

$$\begin{aligned} \tilde{D}(x,y) &= q_R(1+x)(1+y)uv \\ &\quad -2q_R^2(1+x)(1+y)uv \\ &\quad -q_R(1+x)(1+y)(u+v) \\ &\quad -q_R(1+x)(1+y)(uy+vx) - q_R^3 u^2 v^2 \; . \end{aligned} \tag{58}$$

Taking the first and third expression in that list together and exploiting the definitions (56), we obtain

$$\begin{aligned} \tilde{D}(x,y) &= q_R(1+x)(1+y)(uv-u-v) \\ &\quad -2q_R^2(1+x)(1+y)uv \\ &\quad -q_R(1+x)(1+y)(uy+vx) - q_R^3 u^2 v^2 \\ &= q_R(1+x)(1+y)\left\{xy\,\Omega_{RA}\Omega_{RB} - 1\right\} \\ &\quad -2q_R^2(1+x)(1+y)uv - q_R(1+x)(1+y)(uy+vx) - q_R^3 u^2 v^2 \; . \end{aligned} \tag{59}$$

Consequently, we can predict a parameter range in that only less-than-additive effects can be observed:

$$D < 0 \;\; \forall\, q_A, q_B \; : \; q_A\, q_B \le \epsilon' = \frac{1}{\Omega_{RA}\,\Omega_{RB}} \; . \tag{60}$$

The critical value $q_A\, q_B\, \Omega_{RA}\, \Omega_{RB} = 1$ corresponds in the energy space to $E_{RA} + E_{RB} + \Delta G_{A,0} + \Delta G_{B,0} - RT\ln([T_A][T_B]) = 0$ with $\Delta G_{n,0} = G_{n,0,\text{bound}} - G_{n,0}$ and $n = A, B$. More precisely, for $E_{RA} + E_{RB} + \Delta G_{A,0} + \Delta G_{B,0} - RT\ln([T_A][T_B]) > 0$ (< 0) we have $q_A\, q_B\, \Omega_{RA}\, \Omega_{RB} < 1$ (> 1). Consequently, Eq. (60) can alternatively be expressed by

$$D < 0 \;\; \forall\, [T_A], [T_B] \; : \; \Delta G_{A,0} + \Delta G_{B,0} - RT\ln([T_A][T_B]) > -(E_{RA} + E_{RB}) = |E_{RA} + E_{RB}| \; . \tag{61}$$

Note that while we assume that $E_{RA}, E_{RB} \le 0$ holds for activator-induced energy shifts, the expression $\Delta G_{A,0} + \Delta G_{B,0} - RT\ln([T_A][T_B])$ can assume positive or negative values. However, in the limit of small concentrations ($T_n \to 0$) this term becomes positive.
Having examined the case of relatively small transcription factor concentrations, we next focus on the saturation domain: $q_A, q_B \gg 1$. In this case we have $u(x) = \Omega_{RA} x$, $v = y\Omega_{RB}$ and need to take only expressions that are of order 4 into account. Eq. (55) becomes

$$\begin{aligned} \tilde{D}(x \gg 1, y \gg 1) &= \left\{q_R(1-2q_R)\Omega_{RA}\,\Omega_{RB} - q_R\left[\Omega_{RA} + \Omega_{RB}\right] - q_R^3\Omega_{RA}^2\,\Omega_{RB}^2\right\} x^2\, y^2 \\ &= q_R \left\{ \underbrace{(\Omega_{RA}-1)(\Omega_{RB}-1) - 1}_{\text{'first term'}} - 2q_R\Omega_{RA}\,\Omega_{RB} - q_R^2\Omega_{RA}^2\,\Omega_{RB}^2 \right\} x^2\, y^2 \; . \end{aligned} \tag{62}$$

Note that the curled bracket in Eq. (62) can alternatively be expressed by (see main text)

$$W(q_R, \Omega_{RA}, \Omega_{RB}) = (\Omega_{RA}-1)(\Omega_{RB}-1) - \left(1 + q_R\Omega_{RA}\,\Omega_{RB}\right)^2 \; . \tag{63}$$

We have $D < 0$ $(D > 0)$ for $W < 0$ $(W > 0)$. Let us return to Eq. (62). If the expression labeled 'first term' in Eq. (62) is positive and outbalances all other terms in Eq. (62) then we have $\tilde{D} > 0$ and we can observe a greater-than-additive effect caused by the nonlinearities of the transcriptional machinery. Let us illustrate this regime explicitly for the special case $\Omega_{RA} = \Omega_{RB} = \Omega$. In this case, we have

$$\tilde{D} = q_R\Omega x^2 y^2 I \; , \;\; I(\Omega, q_R) = \Omega(1-2q_R) - 2 - q_R^2\Omega^3 \; . \tag{64}$$

The function $I(\Omega, q_R)$ is positive in a particular domain of the Ω-q_R space. The critical boundary line $q_R(\Omega)$ in this space is defined by $I = 0$ and can be determined by solving a quadratic equation. We obtain

$$q_R(\Omega) = \frac{\Omega - 2}{\Omega^2} \tag{65}$$

for $\Omega \geq 2$. The function is shown in Figure 2A and is discussed in detail in the main text.

We close this section by considering the special case in which the relations $q_R \approx 0$, $q_A \Omega_{RA} \gg 1$, and $q_B \Omega_{RB} \gg 1$ hold [6, 7]. Under these conditions, gene expression exhibits relatively small transcription rates but strong activation (see also the main text). Note that we do not necessarily assume that $q_A \gg 1$ and $q_B \gg 1$ holds such that our following considerations are valid for both small and large activator concentrations $[T_A]$ and $[T_B]$ as long as the relations $q_A \Omega_{RA} \gg 1$ and $q_B \Omega_{RB} \gg 1$ are satisfied. We also put $\Omega_{AB} \geq 1$ and to not require $\Omega_{AB} = 1$ ($E_{AB} = 0$). For $q_R \approx 0$, $q_A \Omega_{RA} \gg 1$, and $q_B \Omega_{RB} \gg 1$ the RNA polymerase binding probabilities (21), (27), and (32) read

$$P(x, y) = q_R \frac{x\,\Omega_{RA} + y\,\Omega_{RB} + xy\,\Omega_{RA}\,\Omega_{RB}\,\Omega_{AB}}{1 + x + y + xy\Omega_{AB}} \tag{66}$$

and

$$P(x) = q_R \frac{x\,\Omega_{RA}}{1 + x} \ , \quad P(y) = q_R \frac{y\,\Omega_{RB}}{1 + y} \tag{67}$$

with $x = q_A$ and $y = q_B$. Note that in Eqs. (66,67) we have neglected all terms of order q_R^2 and higher order. We can express Eq. (66) like

$$\frac{P(x, y)}{q_R} = \frac{C + \Omega_{RA}\,\Omega_{RB}\,\Omega_{AB} B}{E + \Omega_{AB} B} \tag{68}$$

with $B = xy > 0$, $E = 1 + x + y > 0$ and $C = x\,\Omega_{RA} + y\,\Omega_{RB} > 0$. From Eq. (67) it follows that

$$\frac{P(x) + P(y)}{q_R} = \frac{C + (\Omega_{RA} + \Omega_{RB}) B}{E + B} \tag{69}$$

such that for $D = P(x, y) - [P(x) + P(y)]$ (see Eq. (47)) we obtain

$$D(x, y) = q_R \frac{\tilde{D}(x, y)}{(E + \Omega_{AB} B)(E + B)} \ . \tag{70}$$

Since $(E + \Omega_{AB} B)(E + B) > 0$ the sign of D is determined by the sign of $\tilde{D}$. A detailed calculation yields

$$\frac{\tilde{D}}{B} = -C(\Omega_{AB} - 1) + E\,\{f_1 f_2 \Omega_{AB} - [f_1 + f_2]\} + B\,\Omega_{AB}\,\{f_1 f_2 - [f_1 + f_2]\} \ , \tag{71}$$

where (to improve readability) we have used the notation $f_1 = \Omega_{RA}$ and $f_2 = \Omega_{RB}$. We are interested in identifying conditions such that a greater-than-additive effect can be observed that is caused by the interplay between the combined, independent activation of the promoter by means of two transcription factors and the nonlinearities of the transcriptional machinery. To this end, we focus on the parameter domain in which $f_1 f_2 > f_1 + f_2$ holds (i.e., we have $\Omega_{RA} \Omega_{RB} > \Omega_{RA} + \Omega_{RB}$). Then, $f_1 f_2 \Omega_{AB} > \Omega_{AB}[f_1 + f_2]$ (because $\Omega_{AB} \geq 1$), which implies

$$\frac{\tilde{D}}{B} \geq -C(\Omega_{AB} - 1) + E\,\{(\Omega_{AB} - 1)[f_1 + f_2]\} + B\,\Omega_{AB}\,\{f_1 f_2 - [f_1 + f_2]\} \ , \tag{72}$$

Since we have $E(f_1 + f_2) > C$ (for definitions of E and C see above), we further conclude that

$$\frac{\tilde{D}}{B} \geq B\,\Omega_{AB}\,\{f_1 f_2 - [f_1 + f_2]\} = B\,\Omega_{AB}\,\{\Omega_{RA}\,\Omega_{RB} - [\Omega_{RA} + \Omega_{RB}]\} > 0 \ . \tag{73}$$

We are left with the problem to determine the parameter domain for which the inequality $\Omega_{RA}\Omega_{RB} > \Omega_{RA} + \Omega_{RB}$ holds. In the Ω_{RA}-Ω_{RB} parameter space the critical line $\Omega_{RA}\Omega_{RB} = \Omega_{RA} + \Omega_{RB}$ defines a hyperbola function of the form

$$\Omega_{RB}(\Omega_{RA}) = \frac{\Omega_{RA}}{\Omega_{RA} - 1} \ , \tag{74}$$

see Figure 4. In summary, we conclude that activation by means of two transcription factors A and B result in a greater-than-additive effect ($D > 0$) for parameters Ω_{RA} and Ω_{RB} that correspond to a point $(\Omega_{RA}, \Omega_{RB})$ located 'above' the hyperbola (74) provided that q_R is sufficiently small and that the products $q_A\,\Omega_{RA}$ and $q_B\,\Omega_{RB}$ (reflecting effective activation levels of the promoter) satisfy $q_A\,\Omega_{RA} \gg 1$ and $q_B\,\Omega_{RB} \gg 1$.

5.1.4 Three-body interactions

As stated in the main text, we examine in this section the analytically solvable problem of gene expression subjected to strong activations at low RNAP concentrations. That is, we assume that $q_A\Omega_{RA} \gg 1$, $q_B\Omega_{RB} \gg 1$, and $q_R \approx 0$ hold (see also the second half of Section 5.1.3). Furthermore, we put $E_{AB} = 0$ ($\Omega_{AB} = 1$) and focus on the role of the energy shift $E_{RAB} \leq 0$ ($\Omega_{RAB} \geq 1$). For $q_R \approx 0$, $q_A\Omega_{RA} \gg 1$, $q_B\Omega_{RA} \gg 1$, $\Omega_{AB} = 1$ the joint and marginal probabilities defined by Eqs. (21), (27), and (32) become

$$P(x, y) = q_R \frac{x\,\Omega_{RA} + y\,\Omega_{RB} + xy\,\Omega_{RA}\,\Omega_{RB}\,\Omega_{RAB}}{(1 + x)(1 + y)} \tag{75}$$

and

$$P(x) = q_R \frac{x\,\Omega_{RA}}{1 + x} \ , \quad P(y) = q_R \frac{y\,\Omega_{RB}}{1 + y} \tag{76}$$

with $x = q_A$ and $y = q_B$ (again we have neglected all terms of order q_R^2 and higher order). The difference (47) can be written like $D = \tilde{D}/[(1 + x)(1 + y)]$ with

$$\tilde{D}(x, y) = \frac{q_R xy}{\Omega_{RAB}} \left\{\Omega^*_{RA}\,\Omega^*_{RB} - [\Omega^*_{RA} + \Omega^*_{RB}]\right\} \tag{77}$$

using the re-scaled parameters $\Omega^*_{RA} = \Omega_{RAB}\,\Omega_{RA}$ and $\Omega^*_{RB} = \Omega_{RAB}\,\Omega_{RB}$. From Eq. (77) it follows that in the two-dimensional space spanned by Ω_{RA} and Ω_{RB} (when Ω_{RAB} is considered as a fixed parameter) there is a critical boundary defined by the hyperbola

$$\Omega_{RB}(\Omega_{RA}) = \frac{1}{\Omega_{RAB}} \left(\frac{\Omega_{RA}}{\Omega_{RA} - 1/\Omega_{RAB}}\right) \ . \tag{78}$$

The hyperbola is shown in Figure 6. Transcription exhibits a greater-than-additive effect (less-than-additive effect) if the parameters Ω_{RA} and Ω_{RB} correspond to a location 'above' ('below') the hyperbola — as indicated in Figure 6 — and if the additional assumptions made earlier hold: $q_R \approx 0$, $q_A\,\Omega_{RA} \gg 1$, $q_B\,\Omega_{RB} \gg 1$, $\Omega_{AB} = 1$ ($E_{AB} = 0$). We would like to point out that Eq. (78) includes the case $\Omega_{RAB} = 1$. In this case, Figure 6 is identical with Figure 4. In this context, it is important to recall the main difference between the derivations leading to Figure 4 and Figure 6. Figure 4 shows the critical boundary line for Ω_{RA} and Ω_{RB} parameters assuming that $\Omega_{AB} \geq 1$ and $\Omega_{RAB} = 1$ hold, whereas Figure 6 was derived under the assumptions $\Omega_{AB} = 1$ and $\Omega_{RAB} \geq 1$. Under the condition $\Omega_{AB} = 1$ and $\Omega_{RAB} \geq 1$ we can determine the separation line between $D > 0$ and $D < 0$ parameter domains (Figure 6). In contrast, under the conditions $\Omega_{AB} \geq 1$ and $\Omega_{RAB} = 1$ we can only determine the boundary line that identifies sufficient conditions for a parameter domain in which $D > 0$ holds (Figure 4).

5.1.5 Saturation case at low RNAP concentrations

For $x, y \to \infty$ (or $x, y \gg 1$) Eqs. (75) and (76) yield the saturation binding probabilities

$$P_{xy,sat} = q_R \Omega_{RA} \, \Omega_{RB} \, \Omega_{RAB} \ , \quad P_{x,sat} = q_R \Omega_{RA} \ , \quad P_{y,sat} = q_R \Omega_{RB} \ . \tag{79}$$

Alternatively, these probabilities can be derived from Eqs. (29,34,36) by putting $q_R \approx 0$ and $\Omega_{AB} = 1$. The corresponding transcriptional activities are given by (see Eq. (16) in the main text)

$$r_{xy,sat} = \beta \, q_R \Omega_{RA} \, \Omega_{RB} \, \Omega_{RAB} \ , \quad r_{x,sat} = \beta \, q_R \Omega_{RA} \ , \quad r_{y,sat} = \beta \, q_R \Omega_{RB} \tag{80}$$

and the basal activity is given by $r_0 = \beta \, q_R$. It has been suggested to use the relations in Eq. (80) to estimate the parameters Ω_{RA}, Ω_{RB}, Ω_{RAB} from r_0, $r_{xy,sat}$, $r_{x,sat}$, and $r_{y,sat}$ or alternatively from the fold changes $r_{xy,sat}/r_0$, $r_{x,sat}/r_0$, and $r_{y,sat}/r_0$ [7]. The advantage of this procedure is that it is not necessary to know the values of β and q_R. We obtain: $\Omega_{RA} = r_{x,sat}/r_0$, $\Omega_{RB} = r_{y,sat}/r_0$, $\Omega_{RAB} = r_{xy,sat}/(r_0 \, \Omega_{RA} \, \Omega_{RB})$.

5.2 Cross-over behavior induced by the dose increase of transcription factors

In this section, we put $q_A = q_B = x$ and $\Omega_{RA} = \Omega_{RB} = \Omega_{Rx}$ (see main text). In this case, the probability (21) reads

$$P_{xx} = \frac{q_R \left(1 + 2x \, \Omega_{Rx} + x^2 \, \Omega_{Rx}^2 \, \Omega_{xx} \, \Omega_{Rxx}\right)}{q_R \left(1 + 2x \, \Omega_{Rx} + x^2 \, \Omega_{Rx}^2 \, \Omega_{xx} \, \Omega_{Rxx}\right) + 1 + 2x + x^2 \, \Omega_{xx}} \tag{81}$$

and describes the RNAP binding probability of a promoter with two identical binding sites. We compare this binding probability with the binding probability of RNAP at a promoter that exhibits only a single binding site for the transcription factor X. In this case, from Eq. (27) it follows that the binding probability of the RNA polymerase is given by

$$P_x = \frac{q_R (1 + x \, \Omega_{Rx})}{q_R (1 + x \, \Omega_{Rx}) + 1 + x} \ . \tag{82}$$

Our aim is to determine the critical value x_c of the cross-over between regimes $P_{xx} < 2P_x$ (less-than-additive) and $P_{xx} > 2P_x$ (greater-than-additive). To achieve this aim, we solve simultaneously the inequalities $P_{xx} < 2P_x$ and $P_{xx} > 2P_x$, which we write in a concise form like

$$P_{xx}(x) \gtrless 2P_x(x) \ . \tag{83}$$

First of all, we express P_{xx} and P_x in terms of

$$P_{xx} = \frac{A}{A + B} \ , \quad P_x = \frac{A'}{A' + B'} \tag{84}$$

with

$$\begin{aligned} A &= q_R \left(1 + 2x \, \Omega_{Rx} + x^2 \, \Omega_{Rx}^2 \, \Omega_{xx} \, \Omega_{Rxx}\right) \ , \\ B &= 1 + 2x + x^2 \, \Omega_{xx} \ , \\ A' &= q_R (1 + x \, \Omega_{Rx}) \ , \\ B' &= 1 + x \ . \end{aligned} \tag{85}$$

Then, we use the function $\chi(z) = (1 - z)/z = -1 + z^{-1}$, which is monotonically decreasing for $z > 0$. We apply this function to both sides of Eq. (83). Since χ is monotonically decreasing we need to change the directions of the inequality signs such that

$$\chi\left(P_{xx}\right) \lessgtr \chi\left(2P_x\right) \ . \tag{86}$$

A detailed calculation shows that $\chi(P_{xx}) = B/A$ and $\chi(2P_x) = (B' - A')/2A'$. After some algebraic transformations we arrive at the expressions

$$2(1{+}2x{+}x^2\Omega_{xx})(1{+}x \, \Omega_{Rx}) \lessgtr \left\{(1 + x) - q_R(1 + \Omega_{Rx} x)\right\} \left(1 + 2x \, \Omega_{Rx} + x^2 \, \Omega_{Rx}^2 \, \Omega_{xx} \, \Omega_{Rxx}\right) \ . \tag{87}$$

5.2.1 Cross-over responses and re-entrant responses caused by nonlinearities of the thermostatistical transcriptional machinery

We solve next Eq. (87) for $\Omega_{xx} = \Omega_{Rxx} = 1$ to study the impact of the energy shift E_{Rx} (Ω_{Rx}). Substituting $\Omega_{xx} = \Omega_{Rxx} = 1$ into Eq. (87), we obtain

$$I(x) = ax^2 + bx + c \lesseqgtr 0 \tag{88}$$

with

$$a = 2 - \Omega_{Rx} + q_R \Omega_{Rx}^2 \ , \quad b = 3 + \Omega_{Rx}(2q_R - 1) \ , \quad c = 1 + q_R > 0 \ . \tag{89}$$

We apply the quadratic formula to $I(x_c) = 0$ to obtain the values of critical concentrations of the activator X for which we see precise additivity in transcriptional output, and consequently switches between less-than-additive and greater-than-additive concentration regions for X. This yields

$$x_c = -\frac{b}{2a}\left(1 \pm \sqrt{1 - \frac{4ca}{b^2}}\right) \ , \tag{90}$$

which can equivalently be expressed by

$$x_c = \frac{-b \pm (\Omega_{Rx} - 1)\sqrt{1 - 8q_R}}{2a} \ . \tag{91}$$

In order to precisely examine these critical values, we first need to distinguish between three broad cases for values of q_R, before breaking down these cases into various subcases. Our general categories are as follows: $q_R = 1/8$, $q_R > 1/8$ and $q_R < 1/8$.

For $q_R = 1/8$, Eq. (91) provides us with exactly one critical value, which is only non-negative (and meaningful as a critical activator concentration) when $\Omega_{Rx} > 4$. For $x < x_c$ transcription is less-than-additive, decreasing in magnitude until additivity at precisely $x = x_c$. Thereafter, transcription becomes less-than-additive again. When we have $\Omega_{Rx} < 4$, we see no non-negative crossover point, and thus we have the same behavior across the full range of concentrations of X, namely a less-than-additive regime. For $\Omega_{Rx} = 4$, the quadratic reduces to a constant, and so we see less-than-additivity for each value of x. Having said that we would like to point out that it is rate that the case $q_R = 1/8$ will occur, since it requires a precise value of q_R. Thus, the remaining two cases $q_R > 1/8$ and $q_R < 1/8$ are our principal concern.

If the normalized RNA polymerase concentration exceeds the threshold concentration $q_R > 1/8$, we have only complex solutions (91) to the quadratic equation (88) with $I = 0$. Consequently, we have no crossover point, and have the same behavior throughout the whole range of activator concentrations. Analysis of Eqs. (88, 91) allows us to conclude that only less-than-additivity is possible for any parameter value Ω_{Rx} and activator doses x. This result is consistent with the result derived earlier in Section 5.1.3 ('Nonlinearities of the thermostatistical transcriptional machinery'), namely, that for $q_R > 1/2$ promoters involving two independently activating transcription factors operate only in a less-than-additive mode.

Our third and final case, where $q_R < 1/8$, is where we see the most interesting behavior. Since $c > 0$ by definition, we need to consider four situations, each yielding different behavior. These are (i) $a > 0$ and $b > 0$,(ii) $a > 0$ and $b < 0$,(iii) $a < 0$ and $b > 0$ and (iv) $a < 0$ and $b < 0$. Each of these pairs of parameter ranges are possible, given the restraints on values of $\Omega_{Rx} > 1$ and $q_R < 1/8$, and does not reach any contradictions for parameter values.

The most interesting behavior is observed when $a > 0$ and $b < 0$. Since Eq. (88) with $I = 0$ reads $ax^2 + bx = -c$ with $c > 0$ we see that the left-hand-side $LHS = ax^2 + bx$ first decays for $x \approx 0$ (because of $b < 0$ and the linear term dominates), then intersects with the horizontal line at $-c$ but finally increases (because of $a > 0$ and the quadratic term dominates) such that there is a second intersection point with the horizontal line at $-c$. In doing so, two real, positive roots, $x_{c,1}$ and $x_{c,2}$ are obtained. For $x < x_{c,1}$ or $x > x_{c,2}$ gene expression is less-than-additive, whereas for $x \in (x_{c,1}, x_{c,2})$ gene expression is greater-than-additive. For an illustration see Figure 9.

5.2.2 Cross-over dose responses induced by three-body interactions

In this section, we put $E_{Rx} = 0$ and $E_{xx} = 0$ ($\Omega_{Rx} = \Omega_{xx} = 1$) and focus on the energy shift E_{Rxx} related to the three-body interaction between RNA polymerase and two molecules of the same transcription factor bound at two different promoter sites A and B. In this case, Eq. (87) reads

$$2(1+x)^2 \lesseqgtr (1-q_R)\left(1+2x+x^2\,\Omega_{Rxx}\right) \ . \tag{92}$$

The critical value x_c for which the equal sign holds (rather than an inequality sign) can be determined by solving a quadratic equation in x. A detailed calculation shows that

$$x_c = \frac{1+q_R}{a}\left(1+\sqrt{1+\frac{a}{1+q_R}}\right) \tag{93}$$

with

$$a = (1-q_R)\Omega_{Rxx} - 2 \ . \tag{94}$$

The critical value (93) only exists for parameters q_R and Ω_{Rxx} such that $a > 0$. That is, for $a \leq 0$ the transcriptional activation exhibits a less-than-additive effect for any relative activator dose x. In contrast, if $q_R < 1$ and $\Omega_{Rxx} > 2/(1-q_R)$ then we have $a > 0$ and for small doses $x < x_c$ transcription with a double binding site promoter shows a less-than-additive effect, whereas for $x > x_c$ the stimulation induced by means of two occupied promoter sites yields greater-than-additive effects, see Figure 10.

Let us discuss briefly how x_c depends on Ω_{Rxx} and q_R. To this end, let us keep q_R constant and vary Ω_{Rxx}. For example, for $q_R = 0.6$ we would move from the location $\Omega_{Rxx} = 10$ (plus sign in Figure 10) to the left towards the critical boundary with $a = 0$ and to the right towards $\Omega_{Rxx} \to \infty$. If we approach the critical boundary with $a = 0$ then we have $a \to 0$ and from Eq. (93) it follows that x_c goes to infinity. That is, the closer the transcriptional machinery operators at the critical boundary, the higher is the transcription factor dose necessary to induce a greater-than-additive response. If we move in the parameter space $\Omega_{Rxx} - q_R$ from the point $q_R = 0.6$, Ω_{Rxx} towards larger values of Ω_{Rxx}, then in the limiting case $\Omega_{Rxx} \to \infty$ we have $a \to \infty$ which implies $x_c = 0$. That is, if the three-body-interaction shifts the RNA polymerase binding energy in a dramatic fashion towards lower energy values then for even the smallest cooperative stimuli $x > 0$ we observe greater-than-additive responses. In short, x_c has a singularity (goes to infinity) at the critical line with $\Omega_{Rxx} = 2/(1-q_R)$ ($a = 0$), decays monotonically as a function of Ω_{Rxx}, and finally approaches zero in the limiting case $\Omega_{Rxx} \to \infty$.

References

[1] Roy BN (2002) Fundamentals of classical and statistical thermodynamics. New York: John Wiley and Sons.

[2] Daune M (1999) Molecular Biophysics. New York: Oxford University Press.

[3] Ackers GK, Johnson AD, Shea MA (1982) Quantitative model for gene regulation by lambda phage repressor. Proc Natl Acad Sci USA 79: 1129-1133.

[4] Shea MA, Ackers GA (1985) The o_r control system of bacteriophage lambda: a physical-chemical model for gene regulation. J Mol Biol 181: 211-230.

[5] Bakk A (2005) Transcriptional activation mechanisms of the Prm promoter of lambda phage. Biophys Chem 114: 229-234.

[6] Bintu L, Buchler NE, Garcia HG, Gerland U, Hwa T, et al. (2005) Transcriptional regulation by the numbers: models. Current Opinion in Gen & Dev 15: 116-124.

[7] Bintu L, Buchler NE, Garcia HG, Gerland U, Hwa T, et al. (2005) Transcriptional regulation by the numbers: applications. Current Opinion in Gen & Dev 15: 125-135.
